# Carboxymethylcellulose/Hyaluronic Acid Hybrid Nanoconjugates: Toward Multifunctional Platforms for Melanoma Skin Cancer Theranostics

**DOI:** 10.3390/gels12100874

**Published:** 2026-09-28

**Authors:** Deborah V. Miranda, Alexandra A. P. Mansur, Herman S. Mansur, Isadora C. Carvalho

**Affiliations:** Center of Nanoscience, Nanotechnology, and Innovation—CeNano2I, Department of Metallurgical and Materials Engineering, Federal University of Minas Gerais—UFMG, Belo Horizonte 31270-901, MG, Brazil; deborahvmiranda@gmail.com (D.V.M.); alexandramansur.ufmg@gmail.com (A.A.P.M.)

**Keywords:** hydrogels, carboxymethylcellulose, hyaluronic acid, hybrid scaffolds, skin cancer, nanotheranostics

## Abstract

Melanoma remains one of the most aggressive forms of skin cancer, characterized by high metastatic potential and risks of tissue trauma and post-surgical recurrence, with limited therapeutic options currently available. While conventional monotherapies often fail to provide comprehensive and efficient treatment, nanoparticle-functionalized hydrogel platforms have emerged as promising multifunctional systems for localized combination cancer therapy, capable of integrating multiple therapeutic modalities, such as chemodynamic therapy (CDT), photodynamic therapy (PDT), and photothermal therapy (PTT), tissue recovery support, antibacterial activity, and image-guided treatment into a single biomaterial. This review examines stimulus-responsive hydrogels based on the anionic polysaccharides carboxymethylcellulose (CMC) and hyaluronic acid (HA), conjugated with nanoparticles for melanoma theranostic applications. It evaluates their design, synergistic mechanisms, and therapeutic potential. It considers how combining CMC and HA overcomes the limitations of individual polymers, such as poor mechanical integrity and rapid degradation, and enhances key functionalities, particularly cell targeting and specificity. It also analyzes how incorporating functional nanoparticles can enable on-demand drug release, diagnostic imaging, immune microenvironment remodeling, and combined phototherapy (PTT/PDT). While HA-based nanotheranostic systems are widely documented, the potential of CMC in melanoma treatment remains largely untapped, despite its proven success in tissue engineering and wound management. Most significantly, the literature currently lacks any bio-nanohybrid platform that integrates CMC and HA, driven by functional hybrid nanoparticles, for targeted melanoma therapy. By identifying this critical gap, this review proposes new research avenues for designing these unified, multifunctional platforms as a highly promising strategy for cutaneous melanoma management.

## 1. Introduction

Skin cancer is a type of cancer characterized by abnormal proliferation of skin cells due to DNA mutations or genetic defects. This neoplasia is broadly classified into two major categories: melanoma and non-melanoma skin cancers, with incidence rates varying across different continents [1]. Although melanoma accounts for only approximately 1% of cases of skin cancer, it is considered the most aggressive and lethal form, being responsible for approximately 80% of skin cancer-related deaths worldwide, largely due to its high metastatic potential [2,3,4]. Furthermore, these rates are projected to increase in the coming years, underscoring the need to develop novel strategies for early diagnosis and more effective therapeutic approaches [1,3].

Conventional dressings currently available for skin cancer patients still present several limitations, including the absence of active immunomodulatory properties and the lack of localized and targeted therapeutic delivery, with the vast majority being unable to simultaneously achieve tumor ablation and tissue regeneration [5,6]. Therefore, there is a strong clinical need for advanced transdermal systems and bifunctional biomaterials that combine antitumor activity with wound-healing and antimicrobial properties. Such a device would enable the eradication of resistant melanoma cells while supporting tissue regeneration and neovascularization [7]. In this context, hydrogels have attracted attention as therapeutic platforms for skin cancer treatment due to their unique chemical structures and properties, including high biocompatibility and bioactivity, form adaptability, injectability, and self-healing [8,9].

Notably, no single polymeric precursor has been reported to simultaneously satisfy all the properties required for an effective skin cancer platform. This is because adequate mechanical robustness, controllable degradation kinetics, tunable stimulus-responsiveness, and biological activity rarely coexist within a single macromolecule. Thus, deliberately mixing two or more precursors has become a central strategy in hydrogel design, allowing the shortcomings of one component to be compensated by the strengths of another. This approach enables rational engineering of platforms with tailored, application-specific activity, achieving multifunctional responses and improving overall performance across a range of medical applications, including tumor therapy and tissue regeneration [8,10,11,12]. Over the last decade, a growing number of studies have explored synergistic combinations of polysaccharides, such as Hyaluronic Acid (HA) and Carboxymethylcellulose (CMC), in hybrid networks aiming to overcome the limitations of individual polymers. In such an approach, one can combine, for example, the bioactivity of HA with the physical stability and enzymatic durability of CMC [13].

In skin cancer treatment, nanotechnology has also been extensively investigated, enabling innovative strategies to improve therapeutic outcomes and enhance treatment efficacy. Examples include advancements in chemotherapy, targeted drug delivery, photodynamic therapy (PDT), photothermal therapy (PTT), and immunotherapy [14,15,16]. In this context, hydrogel-based nanoplatforms have emerged as promising biomaterials for cancer therapy and tissue engineering applications [12].

Despite this growing body of evidence, knowledge regarding CMC- and HA-based hydrogels remains fragmented. Existing studies typically examine each polymer in isolation or explore their combination for applications entirely unrelated to skin cancer. Accordingly, this review synthesizes the state of the art in nanostructured, stimulus-responsive hydrogels utilizing these polysaccharides for melanoma theranostics. While recent notable reviews—such as those by Jin et al. [6], Zhang et al. [17], Alavarse et al. [18], and Wu et al. [12]—have discussed hydrogels for melanoma, stimuli-responsive platforms, or polysaccharide crosslinking chemistry, they treat these subjects independently. Herein, we unify these concepts to rationalize the integration of CMC and HA into a single bio-nanohybrid platform. We evaluate the specific functions, strengths, and limitations of each polymer, justifying their pairing based on targeted suitability rather than outright superiority over other polysaccharide hybrids. Crucially, aiming to guide the development of multifunctional theranostic platforms, we also intend to translate this synthesis into a practical design framework, supported by a crosslinking decision matrix and a rationale for nanoparticle integrations. To our knowledge, no existing review addresses this comprehensive intersection.

## 2. Literature Search Strategy and Scope

This work was conducted as a narrative review with a well-defined scope. Rather than an exhaustive quantitative synthesis of the field, this work aims to provide a critical, integrative overview of CMC- and HA-based stimuli-responsive hydrogels biofunctionalized with nanoparticles for melanoma theranostics. The literature search covered major electronic databases and indexing platforms, including PubMed/MEDLINE, Web of Science, Scopus, and Google Scholar as a supplementary source. Search terms varied by section and included hydrogel, nanogel, scaffold, carboxymethylcellulose, hyaluronic acid, melanoma, skin cancer, wound healing, theranostic, and nanoparticles. To confirm the research gap, we ran a targeted search combining polysaccharide-based hydrogels with nanoparticles and melanoma, but no studies met these criteria. We also screened the reference lists of retrieved articles to identify additional relevant studies.

Eligible sources included peer-reviewed original research articles and review articles published in English. The search excluded editorials, conference abstracts, and non-peer-reviewed materials. We generally excluded studies published before 2018, with preference given to works published within the last five years. Given the limited number of studies in this context that specifically address melanoma, we included selected works on wound healing and other cancer types to provide comparative context and support the rationale for the CMC-HA hybrid design. Because the studies in this review range from material synthesis and physicochemical characterization to preclinical biological assays, each with distinct methodologies and outcome metrics, we did not apply formal quality or risk-of-bias assessments. Instead, studies were selected based on their relevance to the scope, methodological transparency, and inclusion of experimental validation, with their limitations discussed throughout the text.

## 3. Clinical Context: Melanoma Treatment and the Post-Surgical Wound

Skin cancer represents a growing threat to global health systems, with case numbers projected to keep rising over the next two decades [1]. Among its forms, melanoma arises from the dysregulated proliferation of melanocytes, the pigment-producing cells responsible for skin color and for shielding the skin from harmful UV radiation [19]. Its development is driven by the interplay between genetic predisposition and environmental factors, most notably excessive exposure to sunlight and artificial UV radiation [20]. Such a growing burden is already evident in numbers, as in 2022 alone, melanoma accounted for approximately 330,000 new cases and around 60,000 deaths worldwide [21].

Despite advances in melanoma treatment, significant therapeutic challenges remain. Surgery prevails as the primary treatment strategy for malignant melanoma; however, it inevitably causes tissue damage and may impair skin function, while also carrying risks of infection and tumor recurrence [22]. Conventional therapeutic approaches, including chemotherapy and radiotherapy, often have limited efficacy due to the aggressive and metastatic nature of melanoma and are frequently associated with adverse effects [23]. Notably, many anticancer drugs exhibit poor solubility, limited specificity, and systemic toxicity, which can further restrict their therapeutic effectiveness [24]. These challenges may be further exacerbated by immune dysfunction, chronic inflammation, and recurrent infections in cancer patients, potentially contributing to disease progression [25,26].

In modern oncology, two major systemic treatment approaches for advanced or metastatic melanoma are immunotherapy and targeted therapy. Immunotherapies consist of monoclonal antibodies administered via intravenous infusion to reactivate T lymphocytes. In contrast, targeted therapies involve small organic molecules—conventional synthetic chemical drugs such as BRAF inhibitors (dabrafenib, vemurafenib, encorafenib) and MEK inhibitors (trametinib, cobimetinib, binimetinib), formulated for oral administration [2]. Both approaches have transformed the management of advanced melanoma over the past decade. However, despite their clinical success, they both face acquired resistance, which can arise through genetic, epigenetic, signaling, or tumor-microenvironmental mechanisms, ultimately leading to disease progression and treatment failure [6]. Furthermore, these approaches can cause clinically significant adverse effects that affect normal tissues and organs, which may limit treatment tolerability. Immunotherapy, for example, can trigger immune-related adverse events, including colitis, hepatitis, and pneumonitis [20]. Consequently, innovative therapies are urgently needed to evade drug resistance, reduce off-target toxicity, and preserve potent antitumor efficacy against melanoma [27,28].

Although surgical excision is a cornerstone of skin cancer treatment, post-surgical care is equally critical for patient recovery and quality of life [29]. As Jakovija and Chtanova [30] note, the skin serves not only as a physical barrier, but also as an essential immunological defense site. Consequently, restoring this barrier through wound healing is vital; however, the process remains highly complex due to metabolic disruptions within the wound microenvironment [31]. Thus, proper treatment of post-surgical wounds is a fundamental aspect of skin cancer management, especially in patients with systemic conditions that compromise the healing process [26].

Conventional wound dressings for cancer patients present major limitations, such as adhesion-induced tissue damage and poor penetration of topical chemotherapy into the tumor stroma [29]. Furthermore, many UV-responsive hydrogels rely on poorly water-soluble photoinitiators that generate cytotoxic free radicals, damaging healthy tissue and restricting biomedical use [29]. Therefore, advanced hydrogel platforms can serve as complementary, locally acting adjuvant tools. While systemic agents target disseminated and metastatic disease, hydrogel matrices implanted in the surgical bed could provide targeted, sustained local delivery to address residual tumor cells at surgical margins, modulate postoperative inflammation, and support tissue repair. In this context, such platforms may offer a synergistic multimodal approach that could reduce the risk of local tumor recurrence while promoting postoperative wound healing [6,12].

## 4. Nanoparticle-Functionalized Hydrogels for Melanoma Theranostics

Defined as structures with at least one dimension between 1 and 100 nm, nanoparticles (NPs) exhibit a high surface-to-volume ratio and unique physicochemical properties [32]. They are engineered from organic constituents (lipids, polymers, liposomes) or inorganic elements (gold, silver, iron oxide, quantum dots, silica) and are widely utilized as theranostic platforms in oncology [33]. To overcome the shortcomings of conventional treatments, combination therapy has emerged as a promising strategy. By employing two or more modalities (e.g., chemotherapy, PDT, PTT, and immunotherapy) with complementary mechanisms, this approach achieves therapeutic outcomes unachievable by single-modality treatments [1,34,35].

Figure 1 outlines the primary conventional and systemic treatments for melanoma—including chemotherapy and immunotherapy—alongside nanotechnology-based solutions designed to overcome challenges such as drug resistance and off-target toxicity. Additionally, it summarizes major skin cancer types and primary risk factors, providing a comprehensive overview of disease pathophysiology, risk profiles, and the evolving therapeutic landscape [1].

Driven by unique physicochemical and optical properties, NPs are promising tools in cancer therapy and can be engineered for targeted cellular interactions [22,36]. As depicted in Figure 2, NPs leverage internal stimuli (redox, pH) or external triggers (light, heat, ultrasound, magnetic fields) to alter the tumor microenvironment [37], enabling on-demand drug release and boosting photothermal or photodynamic treatments [37]. Moreover, they support simultaneous diagnostic imaging and therapy, creating an integrated theranostic platform.

The high mortality rate of melanoma is primarily driven by its strong metastatic potential and rapid dissemination, making early diagnosis critical for successful patient outcomes. Traditional diagnostic tools often suffer from imprecision, high costs [24], and a strong reliance on examiner expertise. Moreover, definitive confirmation still requires invasive biopsy and histopathological analysis—a workflow plagued by interobserver variability, where specialist panels reclassify up to 17% of initial diagnoses. This diagnostic challenge also leads to a high rate of unnecessary excisions, with some centers performing up to 287 biopsies to identify a single melanoma case [39].

Nanotheranostics have emerged at the forefront of melanoma management by integrating diagnostic imaging and multiple therapeutic agents into a single nanoscale platform [17]. Owing to their high surface-area-to-volume ratio and tunable surface chemistry, these nanostructures can overcome formidable skin barriers to actively target melanoma cells [33,40,41]. Once localized within the tumor stroma, these nanocarriers achieve high accumulation and retention, enhancing cancer cell destruction while mitigating systemic toxicity and side effects in healthy tissues [32,42].

NPs can serve as contrast agents, near-infrared fluorescence sources for deep-tissue imaging, or active targeting agents for diseased cells [14,43,44]. Moreover, they act as versatile nanocarriers for drug delivery, mRNA therapeutics, immunotherapy, tissue regeneration, and cutaneous wound healing [45,46,47]. By contrast, biomaterial scaffolds can provide temporary mechanical support and mass transport, delivering physical and chemical cues with spatiotemporal precision to promote cell adhesion, proliferation, and functional modulation [48]. Consequently, research has increasingly focused on developing cell-inspired biomaterials equipped with drug-loading capacity, stimuli-responsive release kinetics, and cell-membrane camouflage [49].

Among biomaterials, hydrogels stand out as one of the most extensively studied and versatile classes, categorized by origin (natural, synthetic, or hybrid) and crosslinking mechanism (physical or chemical networks) [50]. Their defining feature is a hydrophilic polymeric network capable of absorbing and retaining vast amounts of water or biological fluids while maintaining structural integrity. Because crosslinking type and synthesis conditions govern their final properties [18], hydrogels can be tailored to closely mimic the extracellular matrix (ECM) and support cellular interactions within a biocompatible, mechanically tunable environment [50], making them ideal base materials for scaffold construction. Beyond structural adaptability, they have garnered extensive biomedical interest for traits such as injectability and self-healing capacity, the latter driven by reversible chemical bonds that allow the material to withstand physiological mechanical stress and spontaneously repair internal damage [9]. These features are also central to minimizing systemic toxicity while maximizing local therapeutic efficacy [33,51]. Furthermore, their porous architecture facilitates the diffusion of bioactive molecules, enabling sustained local drug release and fostering a favorable microenvironment for tissue regeneration [50].

## 5. Anionic Polysaccharides as Building Blocks: CMC and HA

### 5.1. Polysaccharides as Hydrogel Building Blocks

Polysaccharides are an important class of macromolecules composed of repeating carbohydrate units. They are among the most widely used building blocks in hydrogel design because of properties such as bifunctionality, therapeutic selectivity, self-assembly capacity, and stimuli responsiveness [26,52,53]. Their molecular chains carry several readily modifiable functional groups that form intra- and intermolecular interactions, such as hydrogen bonding, hydrophobic effects, and electrostatic interactions, which drive chain entanglement and ultimately give rise to the three-dimensional crosslinked networks discussed above [54]. These same chemical groups also allow polysaccharides to be functionalized at different sites, tailoring their structure to produce hydrogels with improved physicochemical and biological properties [55].

Categorizing polysaccharides according to their ionizable functional groups is fundamentally important: neutral polysaccharides are characterized by abundant hydroxyl groups (-OH); cationic polysaccharides incorporate amino groups (-NH_2_) subject to protonation-induced positive charges; and anionic polysaccharides contain acidic functionalities, notably carboxyl groups (-COOH), which ionize to form negative charges [56]. The high density of these anionic groups facilitates robust hydration and water retention, establishing hydrogel networks that support cell viability, growth, and tissue repair. Moreover, their susceptibility to enzymatic degradation into biocompatible, non-toxic metabolites underlines their high utility in long-term biomedical interventions [57].

### 5.2. Carboxymethylcellulose and Hyaluronic Acid

Carboxymethylcellulose is an anionic, semi-processed biopolymer derived from cellulose by partially substituting hydroxyl groups (-OH) on its glucopyranose rings with carboxymethyl groups (-CH_2_-COOH) [58]. It is a non-toxic, biodegradable, water-soluble, environmentally friendly, low-cost compound. In addition, this polysaccharide adheres effectively to wound sites, absorbs exudates (fluids), and maintains a favorable healing environment; it is also used as a multifunctional organic shell to cap ligands and stabilize inorganic nanoparticles [38]. Previous research has shown that CMC can provide a biocompatible surface for developing hydrogels for biomedical applications, such as skin substitutes, drug delivery systems, and bioimaging platforms for treating various diseases, including cancer [38]. Figure 3A represents the chemical structure of CMC.

Hyaluronic acid is a natural, linear, anionic mucopolysaccharide that serves as a major constituent of the native extracellular matrix, linking tissues and the dermis. Involved in diverse biological processes, HA exhibits molar-mass-dependent properties that regulate angiogenesis, tissue regeneration, and inflammatory responses [55]. As depicted in Figure 3B, HA features versatile reactive functional groups—including hydroxyl, carboxyl, and acetyl groups—that enable chemical modification, alongside intrinsic biocompatibility, biodegradability, and non-immunogenicity [59]. Figure 4 provides a comprehensive overview of the chemical versatility of CMC and HA for theranostic platform development, highlighting key reaction sites derived from literature-reported strategies for chemical modification, functionalization, and crosslinking. Specifically, the figure illustrates how these reactive moieties, predominantly carboxyl and hydroxyl groups, are systematically exploited to engineer advanced multifunctional architectures.

### 5.3. Hybrid Systems with CMC and HA for Melanoma Management

Researchers have widely investigated combining CMC and HA with other natural or synthetic polymers to form hybrid hydrogel networks, offering significant physicochemical, mechanical, and biological benefits for melanoma management, including tissue regeneration, wound care, and tumor cell elimination [5,72,73,74,75,76,77,78]. Research on hybrid hydrogels for cutaneous wound treatment increasingly focuses on blending CMC and HA with complementary polymers to overcome individual shortcomings and achieve synergistic performance [79].

To address the intrinsic limitations of CMC alone, Jeong et al. [74] investigated a wound-healing hydrogel composed of gelatin and oxidized carboxymethylcellulose (OCMC) loaded with fucoidan, a sulfated marine polysaccharide. In this design, gelatin served as an ECM-mimetic protein base, while fucoidan acted as a bioactive therapeutic agent, inhibiting nitric oxide (NO) production and stimulating de novo collagen synthesis by fibroblasts [74]. To counteract the poor mechanical strength and thermal instability of pure gelatin, the authors incorporated CMC. By oxidizing CMC to generate reactive aldehyde groups, they facilitated spontaneous condensation with gelatin’s free amine groups, establishing a stable three-dimensional network without toxic synthetic crosslinkers. Because these imine bonds are reversible, the resulting network balanced structural integrity with dynamic self-recovery, enabling the gel to adapt to mechanical stresses within the wound bed. In a murine full-thickness model, this optimized formulation accelerated wound closure by the third day and achieved complete re-epithelialization within 14 days [74]. Nonetheless, fucoidan-based formulations present certain challenges: their high swelling capacity can lead to an initial burst release of bioactive cargo, and structural variations across seaweed species can complicate standardization and batch-to-batch reproducibility [74]. Ultimately, these mechanical adaptations and self-healing properties, combined with effective exudate management, position CMC-based hydrogels as safe and promising wound-care options for melanoma patients. Furthermore, this same CMC–fucoidan combination has been explored for minimally invasive adjuvant melanoma therapy as a dissolvable microneedle patch, where CMC provides the requisite mechanical support while fucoidan acts as the primary antitumor agent [76].

On the other hand, despite its exceptional biocompatibility and water-retention capacity, HA forms hydrogels that often exhibit mechanical weakness, high solubility, and rapid or uncontrolled degradation in physiological environments [78]. As a native macromolecule, HA is highly susceptible to enzymatic degradation by hyaluronidases, which can further compromise its structural stability [55,69]. To address such drawback, Zhao et al. [72] crosslinked thiolated gelatin with dopamine-modified HA and loaded the resulting bioabsorbable scaffold with doxorubicin-carrying, reduction-sensitive polymeric nanomicelles: once implanted in the post-resection bed, the porous matrix absorbs the wound exudate and physically traps the residual B16F10 cells that escaped surgery, which are then eliminated through RGD-mediated endocytosis of the nanocarriers, while hyaluronic acid contributes to tissue repair by regulating cell proliferation, adhesion and differentiation; the platform was nevertheless designed for antitumor therapy alone, without functions such as bleeding control or skin reconstruction. Schmid et al. [75] followed the opposite rationale and used a printable alginate/HA/gelatin bioink, in which hyaluronic acid provides CD44 binding and gelatin provides integrin binding, to biofabricate vascularized metastatic melanoma models for disease modeling and drug screening rather than a therapeutic product. Other combinations associate phototherapy with tissue regeneration [5,73,77] and are analyzed individually in Section 7.

Taken together, these studies show that combining CMC or HA with a second polymer improves the physicochemical and mechanical properties and responsive behavior of the gels. Read comparatively, however, they also reveal a consistent division of labor directly relevant to the design proposed here: in each of these hybrids, one polysaccharide carries the tumor- or tissue-directed function, while the partner supplies structure. Gelatin mimics the dermal extracellular matrix but is mechanically weak and unstable at physiological temperature, and in both systems in which it appears it had to be rescued by a polysaccharide—by oxidized CMC, whose aldehyde groups condense with its free amines, in the dressing of Jeong et al. [74], and by dopamine-modified HA in the bioabsorbable scaffold of Zhao et al. [72]. Schmid et al. [75] state the division explicitly, assigning CD44 binding to HA and integrin binding to gelatin within an alginate-based printable network.

Two key properties account for this recurrence and distinguish the CMC–HA pair from other hybrid combinations reported for melanoma. First, cellular targeting is intrinsic to the matrix rather than artificially grafted: HA is the endogenous ligand for CD44, a receptor heavily overexpressed in melanoma. Thus, an HA-based network displays the recognition motif natively at a density dictated by formulation rather than a separate conjugation step. Conversely, integrin binding is not selective for melanoma, and neither chitosan nor alginate offers a comparable receptor-level interaction. Second, electrostatic compatibility with the nanoparticle phase is uniquely favorable. Both CMC and HA are polyanions across physiological and mildly acidic pH ranges; the identical carboxylate groups engaging CD44 also cap and anchor metal, metal-oxide, and semiconductor nanoparticles. Thus, network crosslinking and nanoparticle stabilization rely on a single, unified chemistry. In contrast, polycation–polyanion pairs (e.g., chitosan–alginate) form networks through the electrostatic complexation of those precise functional groups, rendering them unavailable to stabilize anionic nanoparticles.

Importantly, this rationale is founded on functional suitability rather than absolute superiority, and trade-offs must be acknowledged. Quaternized chitosan, for instance, contributes intrinsic antimicrobial activity that neither CMC nor HA possesses (as seen in the self-healing dressing in Section 6.1), meaning a CMC–HA platform must derive its antimicrobial behavior from the nanoparticle phase. Similarly, gelatin supplies cell-adhesive sequences and protease-susceptible degradation sites, while HA is far from an inert scaffold, as its molecular weight and degree of chemical modification govern both CD44 engagement and angiogenic responses (Section 8.1 and Section 8.2). The CMC–HA combination is not an inherently superior polymer, but a unique pairing among those reviewed in which receptor-level targeting, anionic nanoparticle compatibility, enzymatically regulated degradation, and established wound-dressing performance all derive from the same two macromolecules. To our knowledge, no existing system unites both polysaccharides into a single nanoparticle-functionalized network for melanoma. It is this convergence of functions—rather than the standalone novelty of the polymer combination—that motivates the design framework developed in Section 8. To avoid redundancy, Table 1 (Section 7.3) compares the composition, crosslinking chemistry, therapeutic modality, validation stage, outcomes, biosafety evidence, and limitations of these systems.

## 6. HA and CMC-Stimuli-Responsive Hydrogels in Cancer Management and Wound Healing

The inherent stimulus responsiveness of polysaccharides such as HA and CMC becomes a major practical advantage upon forming a hydrogel network. These “smart” platforms can sense physical and chemical changes in their surrounding environment—such as variations in pH, temperature, or enzyme concentration—and respond accordingly by releasing therapeutic agents at controlled concentrations directly to pathological sites while minimizing exposure to healthy tissues [80,81].

Hydrogels can be strategically engineered to respond to internal cues, such as ionic strength, reactive oxygen species (ROS), or specific enzymes, or external triggers, to mention magnetic fields, electric fields, ultrasound, or temperature [82]. Researchers achieve this responsiveness by incorporating functional compounds, introducing dynamic or cleavable crosslinks tailored to biological conditions, or integrating functional nanoparticles sensitive to external stimuli like magnetic fields, light, or heat. These triggers induce structural alterations—such as conformational shifts, phase transitions, swelling changes, or permeability adjustments, thereby enabling precise, on-demand drug delivery [53,83,84].

Among these design strategies, the choice of crosslinking approach is particularly influential, as it dictates stimuli-responsive behavior while governing broader structural and biological performance. Salma-Ancane et al. [85] highlight this dual role by synthesizing hyaluronic acid and ε-polylysine (ε-PL) hydrogels via distinct physical and chemical methods. While both hydrogel types exhibited rapid swelling, long-term stability, and comparable stiffness, their properties diverged based on the crosslinking mechanism. Physical crosslinking relied on electrostatic interactions between anionic HA and cationic ε-PL, whereas chemical crosslinking explored EDC/NHS coupling. Chemically crosslinked networks featured lower crosslinking density and higher porosity, enabling significantly greater liquid absorption and swelling. Furthermore, these chemically crosslinked hydrogels showed reduced antibacterial activity because fewer free amine groups were available to interact with bacterial cell walls, yet they exhibited superior cytocompatibility [85]. Most of all, this study underscores the importance of tailoring the crosslinking method to the intended application.

### 6.1. CMC and HA Stimuli-Responsive Hydrogels in Wound Healing

The tunability of polysaccharides such as HA and CMC has driven their application across diverse biomedical fields, particularly in developing smart, stimulus-responsive hydrogels for advanced wound management. For instance, Yang et al. [86] engineered a self-healing, antibacterial hydrogel composed of aldehyde-modified hyaluronic acid and fungal-derived quaternized chitosan (QAC), utilizing 3,3′-dithiobis(propionyl hydrazide) (DTP) as a crosslinking agent to form both dynamic chemical bonds and physical interactions. Driven by fast reaction kinetics and reversible networks, including dynamic covalent bonds (hydrazone and/or disulfide linkages) and electrostatic interactions, the hydrogels exhibited rapid gelation and excellent self-healing capabilities [86]. This dynamic nature allows the material to self-repair: while these bonds break under mechanical stress, they spontaneously reform when damaged surfaces re-contact, restoring structural integrity and function. While cytocompatible and promising for wound dressings, the system suffered from limited mechanical strength due to its dynamic crosslinks, a defect compounded by QAC, which reduced the stress–strain at break. The authors also disclosed that QAC imparted type-dependent antibacterial efficacy, achieving complete inhibition (~100%) against Gram-negative *E. coli* but lower performance (75.9%) against Gram-positive *S. aureus*. Consequently, optimizing such platforms will require replacing or supplementing QAC with alternative broad-spectrum antimicrobial agents—such as antimicrobial peptides or inorganic nanoparticles—to boost bactericidal activity without compromising mechanical performance [86].

Seeking to improve wound treatment while maintaining effective antibacterial activity, Liu and colleagues [87] developed a multifunctional hydrogel termed HTFC, which incorporates HA and calcium peroxide nanoparticles. These nanoparticles not only boost antibacterial efficacy against both Gram-positive and Gram-negative strains via chemodynamic therapy, but also mitigate hypoxia—critical for supporting cell proliferation and angiogenesis within the wound bed—while enhancing overall hydrogel stability. Overall, this work highlights nanoparticle incorporation as a powerful multifunctional strategy for treating infected wounds, enabling simultaneous bactericidal activity, microenvironmental modulation, and accelerated healing. However, as with other calcium peroxide-based systems, this therapeutic benefit relies on tightly controlling H_2_O_2_ and ROS generation rates, since excessive release can damage healthy tissue [87].

In contrast to Yang’s reliance on dynamic, reversible bonds, B. Huang et al. [88] developed a system centered on covalent crosslinking to ensure structural integrity and mechanical stability. The authors synthesized an HA-based hydrogel for wound repair, designed to combat bacterial infections through PDT via ROS generation by combining methacrylated hyaluronic acid (HAMA), a modified porphyrin photosensitizer (TPP), and dopamine methacrylamide (DMA). In this formulation, functionalization introduced methacrylate groups into HA and dopamine, providing chemical anchoring points for subsequent crosslinking while imparting structural stability and strong tissue adhesion [88]. Furthermore, the hydrogel produced significantly more ROS upon laser irradiation than an equivalent solution of TPP nanoparticles encapsulated in an amphiphilic copolymer. The authors attributed this difference to self-quenching of porphyrins within the standalone nanoparticles, demonstrating that hydrogel integration prevents aggregation and enhances efficiency [88]. Ultimately, this approach yielded a smart, synergistic wound dressing capable of eliminating bacterial infections, adhering firmly to damaged skin, and providing robust biological support for tissue regeneration [88].

### 6.2. CMC and HA Stimuli-Responsive Hydrogels in Cancer Management

Several studies have explored incorporating nanoparticles, functionalization, and advanced crosslinking strategies to develop more effective therapeutic approaches and improve outcomes in cancer management. For instance, Ali and collaborators [89] developed near-infrared (NIR)-responsive carboxymethyl cellulose hydrogels containing thioketal linkages for on-demand drug delivery in cancer therapy. This crosslinker serves two critical functions: it confers ROS-responsiveness and features tetrazine end groups that react with polymer precursors chemically modified with complementary reactive groups. Crosslinking proceeds via an ultrafast Inverse Electron Demand Diels-Alder (IEDDA) click reaction, which generates nitrogen (N_2_) gas as a by-product. This outgassing creates porosity and microbubbles within the network, enhancing drug loading and release. Furthermore, NIR light acts as a remote trigger to release doxorubicin (DOX) and boost antitumor efficacy through chemo-photothermal therapy, facilitated by the encapsulated photosensitizer indocyanine green (ICG), which converts light into localized heat to disrupt the hydrogel matrix. The choice of crosslinker was fundamental to the platform’s success, enabling precise on-demand responsiveness. Through chemo-photothermal combination therapy and in vitro testing, the hydrogel demonstrated high efficacy, safety, and highly controlled release kinetics [89]. Nevertheless, comprehensive in vivo studies remain essential to fully establish its translational potential.

Shi et al. [54] developed an injectable hyaluronic acid-based hydrogel loaded with DOX for breast cancer treatment and metastasis prevention. The HA chains were first modified by conjugating methacrylohydrazide (MHB) molecules via amidation, yielding unsaturated double bonds and amide linkages. The modified precursor was then crosslinked with a sulfhydryl-based agent via a Michael addition reaction to form the hydrogel network, into which DOX was dissolved to create the therapeutic matrix. By tailoring HA modification and selecting an appropriate crosslinking mechanism, the authors engineered a biomaterial responsive to thermal stimuli and pH-dependent swelling, enabling refined control over release kinetics and reduced systemic toxicity [54]. Although the platform showed promising results with isolated DOX delivery, its potential could be expanded by incorporating synergistic drug combinations—an approach particularly relevant for metastatic breast cancer. Additionally, future investigations could explore integrating functional nanoparticles into the hydrogel network to boost therapeutic efficacy and introduce multi-modal functionalities.

Zheng et al. [64] developed a multifunctional CMC-based nanohydrogel incorporating DNA-templated CdTeSe quantum dots (QDs) for tumor theranostics, with Figure 5 schematically illustrating its design, synthesis, and mechanism of action. In this architecture, CMC serves as the structural scaffold, while the QDs function as fluorescent probes for diagnostic imaging and monitoring. Rather than relying solely on gradual framework degradation, the nanohydrogel achieves on-demand, intelligent drug release via a dual-stimulus disassembly mechanism triggered by elevated levels of glutathione (GSH) and specific microRNAs (miRNAs) within the tumor microenvironment, ensuring targeted doxorubicin delivery [64]. Whilst the system successfully protected cargo from degradation, enabled active tumor targeting, and provided intelligent release kinetics, heavy metal toxicity remains a significant barrier to clinical translation despite low short-term cellular toxicity. Furthermore, the authors reported challenges in tracking the nanohydrogel in vivo via intravenous fluorescence imaging, likely attributable to insufficient QD concentration. Consequently, this work underscores the critical need for alternative nanoparticle formulations featuring reduced toxicity and enhanced fluorescence efficiency to enable precise oncological diagnostics [64].

Recent literature has investigated a wide range of nanomaterials for cancer diagnosis and treatment, including conventional metallic and magnetic nanoparticles as well as semiconductor quantum dots. However, the clinical translation of nanotheranostic platforms depends not only on imaging and therapeutic performance but also on biological safety. Achieving an optimal balance among high optical brightness, therapeutic efficacy, and biocompatibility remains a critical challenge in developing next-generation nanotheranostics, underscoring the need for continued research [90,91].

Wang et al. [92] engineered an injectable hydrogel containing all-trans retinoic acid (ATRA) and gold nanoparticles designed to prevent breast cancer recurrence and metastasis by directly targeting tumor stem cells. Specifically, the researchers developed ATRA-loaded HA micelles to induce the differentiation of cancer stem cells (CSCs) into regular cancer cells, rendering them far more vulnerable to elimination via localized hyperthermia generated by the gold nanoparticles under laser stimulation. In this formulation, HA was chemically modified by conjugating it with oleic acid via cystamine linkages to form stable micelles that gradually release and bind to CD44 receptors on tumor cells. Thus, hyaluronic acid plays a pivotal role by enabling highly selective targeting of cancer stem cells overexpressing the CD44 receptor. While this targeting approach yielded highly promising therapeutic outcomes, treating deep-seated tumors with PTT remains challenging in clinical settings due to the limited tissue penetration depth of infrared light [33,93].

In oncology, polysaccharide-based systems have been extensively validated across various cancer models, with previous studies independently demonstrating advances in sustained drug delivery, tumor control, diagnostics, and combination therapies [55,67,94]. Despite this broad body of work, CMC- and HA-based systems investigated specifically for melanoma nanotheranostics remain scarce. While the individual properties of CMC and HA hydrogels—such as intrinsic tumor targeting, anticancer and antibacterial activities, and high nanoparticle-loading capacity—are highly promising, a significant research gap persists regarding their direct application to melanoma. Consequently, the following sections examine the limited yet growing body of research dedicated to treating this aggressive cutaneous neoplasm, which is frequently complicated by ulceration, high metastatic potential, and severe infection risks.

## 7. CMC and HA Stimuli-Responsive Hydrogels in Melanoma Management

Treating melanoma is inherently challenging due to complex biological, genetic, and anatomical factors, establishing it as one of the most aggressive and treatment-resistant malignancies known. Key factors driving this complexity include a high propensity for rapid metastasis, extreme genetic heterogeneity, intrinsic and acquired treatment resistance, an immunosuppressive tumor microenvironment, and clinical hurdles such as the persistence of residual tumor cells [2,20,95]. Consequently, developing alternative therapeutic interventions remains essential to bypass resistance mechanisms, minimize adverse side effects, and maintain robust therapeutic control [27,28]. In this context, smart hydrogels represent a highly promising strategy for cutaneous cancers like melanoma, as they overcome conventional limitations through advanced targeting, enhanced safety profiles, and multi-functional performance [55,69].

### 7.1. Nanoparticle-Functionalized CMC Hydrogels for Melanoma Management

Despite the widespread use of nanoparticle-functionalized hydrogels in wound healing and oncology, literature specifically combining CMC with nanoparticles for melanoma treatment remains notably scarce, with few studies directly validating CMC-based nanostructured hydrogels against melanoma cell lines. In a pioneering study, Capanema et al. [25] designed and synthesized hybrid hydrogels composed of CMC conjugated to DOX alongside silver nanoparticles (AgNPs), which serve as dual antitumor and antimicrobial agents by inducing oxidative stress and apoptosis. The AgNPs act synergistically with doxorubicin, substantially amplifying human melanoma cell death (A375 line). While the pristine polymeric hydrogel exhibited no inherent cytotoxicity, the drug-loaded hybrid formulations demonstrated a protective release profile. Given that skin cancer patients frequently present with compromised immunity and ulcerated, infection-prone lesions, the inclusion of AgNPs provided vital antimicrobial protection [25]. Crucially, carboxymethylcellulose functioned far beyond a passive structural matrix: it played an indispensable, multifunctional role by promoting the in situ reduction of silver ions, acting as a capping agent via carboxylate groups to stabilize the AgNPs, facilitating doxorubicin entrapment and controlled release, and ensuring overall biocompatibility. Furthermore, modulating the density of CMC carboxylate groups (comparing degrees of substitution of 0.8 versus 1.2) enabled precise tuning of network swelling and release kinetics. Although the hydrogel demonstrated excellent physicochemical and biological properties in vitro, the authors emphasized the necessity for robust in vivo validation to account for the structural and functional complexity of human skin [25].

Priya and collaborators [63] developed an innovative nanostructured CMC-based polymeric gel for targeted topical melanoma treatment. The system features a smart core–shell architecture in which the core consists of a self-assembled carboxymethyl cellulose and casein (CA) nanogel, while the shell comprises an ultrathin outer coating built from alternating layers of positively charged casein and folic acid = as a negatively charged ligand. The outermost folic acid layer acts as a targeting motif, as melanoma cells (MEL-39 line) overexpress folate receptors that selectively recognize and internalize the nanogel. CMC plays a vital role in structural cohesion and physical stability: as an anionic polysaccharide, it engages in direct electrostatic interactions with cationic casein to form a highly integrated, three-dimensional porous network that drastically improves the aqueous solubility of curcumin. Curcumin is a highly promising natural anticancer compound whose clinical utility is otherwise severely restricted by extremely low water solubility and rapid in vivo degradation. The authors designed this nanoscale formulation to overcome skin barriers, enhance tissue permeability, facilitate cellular endocytosis, and stabilize curcumin, yielding a smart-release system significantly more effective at inducing melanoma cell death (MEL-39 line) than the free drug. Nevertheless, this platform lacks in vivo evaluation in complex living organisms, and its long-term storage stability remains unassessed [63].

Beyond these systems, literature describing nanostructured or nanoparticle-incorporated CMC hydrogels specifically for melanoma treatment remains exceptionally sparse. Nevertheless, studies by the same research group as Capanema et al. [25] describe localized therapeutic platforms—specifically skin patches and dressings—composed of CMC crosslinked with citric acid and loaded with doxorubicin [65,66]. Incorporating DOX into these CMC matrices successfully mitigated the severe, uncontrolled systemic toxicity associated with the free chemotherapeutic agent, while ensuring sustained drug delivery and cellular internalization via active endocytosis. Although these earlier iterations did not incorporate nanoparticles, both formulations yielded highly promising outcomes against melanoma, laying a solid foundation for advancing nanostructured platforms in this field.

The study by Silva et al. [76] describes the development of dissolvable polymeric microneedle patches composed of CMC and fucoidan—a bioactive polysaccharide derived from brown algae that acts as the active agent against tumor cells—for the minimally invasive, adjuvant treatment of melanoma. Fucoidan exerted concentration-dependent cytotoxic and antiproliferative effects on A375 melanoma cells through apoptosis induction, cell cycle arrest, and the inhibition of tumor migration. Meanwhile, CMC formed the body and rigid shafts of the needles, providing mechanical strength comfortably above the minimum force of 0.15 N required to penetrate the stratum corneum, while dissolving completely within approximately 12 min to ensure the rapid release of fucoidan into deeper skin layers. Cell viability was reduced by approximately 83% in conventional two-dimensional cultures and by about 56% in a bioprinted three-dimensional tumor model after 48 h, establishing the biomaterial as a promising strategy to prevent local recurrence following surgical excision [76]. The lower sensitivity observed in the three-dimensional model reflects the greater architectural and physiological complexity of architectures that faithfully mimic the tumor microenvironment. Nonetheless, the researchers emphasize that in-depth evaluations of the patch’s biocompatibility and systemic toxicity remain essential. Furthermore, because fucoidan alone may be insufficient against highly aggressive melanomas, the authors suggest investigating co-administration of fucoidan with complementary chemotherapeutic drugs or bioactive compounds within a unified microneedle platform [76].

CMC hydrogels functionalized with metal oxides have demonstrated potent cytotoxicity against skin cancer cells [78]. In research conducted by Enoch and Somasundaram [78], a CMC-based drug-delivery platform was engineered with exceptional water-retention capacity and biodegradability, incorporating Carbopol as a co-polymer to confer structural stability and eliminate the need for potentially toxic synthetic crosslinkers. Furthermore, the authors utilized a phytotherapeutic active ingredient (*Justicia adhatoda*) alongside manganese oxide nanoparticles, which aided in gel stabilization, modulated release kinetics, and served as active agents against bacterial pathogens and tumor cells. In summary, the hydrogel achieved controlled drug delivery, antimicrobial efficacy, and targeted anticancer activity. Nevertheless, the study did not address the in vivo behavior or long-term toxicity profile of the manganese oxide nanoparticles—an essential consideration given the known potential for off-target oxidative stress associated with metal-oxide nanomaterials [78].

A study by Behbood and colleagues [68] described a sophisticated platform for the topical treatment of skin cancer, consisting of self-assembling, nanostructured polymeric micelles based on deoxycholic acid incorporated into a CMC hydrogel support. The system was designed for dual drug delivery: releasing the chemotherapeutic agent 5-fluorouracil (5-FU) encapsulated within the micelles, alongside the anti-inflammatory steroid dexamethasone incorporated directly into the CMC matrix to mitigate 5-FU-induced skin irritation. Here, CMC served as a structural delivery vehicle, providing a protective coating effect and acting as a pH-sensitive release trigger. The system demonstrated pH-modulated drug release driven by microenvironmental acidity and exhibited potent in vitro anticancer efficacy against human epidermoid carcinoma cells (A431). However, the researchers emphasize that further in vivo investigations are required to fully evaluate actual skin permeation, local pharmacokinetics, and therapeutic efficacy. Concurrently, CMC systems incorporating NIR-responsive gold nanorods, developed by Chang et al. [92], have yielded promising results in colon cancer treatment, demonstrating high anticancer efficacy. Similarly, Ali et al. [89] reported a smart CMC hydrogel that uses indocyanine green—an organic compound that absorbs NIR light to generate heat and reactive oxygen species—effectively combining photothermal and therapeutic modalities for cervical cancer management.

### 7.2. Nanoparticle-Functionalized HA Hydrogels for Melanoma Management

A promising histidine-functionalized HA nanogel (HA-His) was developed in a study led by Pan et al. [96] aiming at melanoma theranostics. In this work, the system was self-assembled in a single step using manganese ions as reversible crosslinkers and loaded with DOX alongside the photosensitizer Chlorin e6 (Ce6). Beyond forming the three-dimensional physical matrix that houses the therapeutic cargo, HA is displayed on the outer surface of the nanogel, enabling active tumor targeting via specific binding to overexpressed CD44 receptors. Together, these features empower the platform to integrate active targeting, responsive drug release, and laser-activated PDT. Further, manganese-ion-enabled magnetic resonance imaging (MRI) provides diagnostic capability, enabling in vivo tracking of nanogel distribution and confirming high accumulation within tumor tissue [96]. However, the study encountered limitations: the generation rate of singlet oxygen (a reactive oxygen species) was somewhat lower than that of free Ce6 in solution, attributable to partial aggregation of Ce6 molecules upon encapsulation within the polymer matrix. Furthermore, future investigations must validate whether larger organisms can safely eliminate or tolerate this nanostructure over extended periods.

Like Pan et al. [96], other researchers have utilized the photosensitizer Chlorin e6 (Ce6) to engineer hydrogels for melanoma treatment, each employing a distinct strategy [5,97]. Specifically, Zhang et al. [97] modified cerium oxide (ceria) nanoparticles with HA and covalently linked Ce6 to construct a nanostructured phototherapeutic complex capable of sustained oxygen generation. Figure 6 illustrates the general synthesis scheme—encompassing nanoparticle formation, oxidation state transitions, and thermosensitive gelation—alongside the system’s underlying mechanism of action. Upon administration at the tumor site, calcium peroxide progressively reacts with infiltrated water to generate hydrogen peroxide, which the nanoceria (HCeC) subsequently catalyzes to release molecular oxygen, alleviating tumor hypoxia. Once the microenvironment is reoxygenated and loaded with targeted Ce6, 660 nm laser irradiation excites the photosensitizer to generate highly cytotoxic ROS and singlet oxygen for melanoma eradication. In this architecture, HA functions as both a surface stabilizer and a targeting ligand, preventing nanoparticle aggregation and ensuring excellent aqueous dispersion. Consistent with Pan et al.’s platform [96], HA further promotes selective cellular uptake via overexpressed CD44 receptors on tumor cells, ensuring high therapeutic specificity.

Zhang et al. [5] developed an innovative HA-based hydrogel microneedle system designed for simultaneous melanoma ablation and skin regeneration. In this architecture, Ce6 is chemically conjugated to a multifunctional nanomaterial comprising carbon nanotubes, calcium peroxide, and a tannic acid-Fe complex (CCa@TF/Ce6) to form microneedle tips that penetrate the epidermis and deliver the therapeutic payload. This CCa@TF/Ce6 nanocomposite drives tumor destruction by synergizing immune activation, PTT, and PDT. Concurrently, the microneedle base—composed of HA and polyvinyl alcohol (PVA)—remains on the skin surface to facilitate post-treatment tissue repair. Demonstrating high efficacy and a favorable safety profile, the system achieved near-complete tumor suppression in melanoma xenograft models [5]. Furthermore, it successfully overcame a primary limitation of PDT—insufficient singlet oxygen production under hypoxic conditions—by employing a self-oxygenating strategy: the incorporated calcium peroxide and catalytic complex generate in situ oxygen to alleviate hypoxia and fuel Ce6-mediated ROS production [5].

Similarly addressing the obstacle of tumor hypoxia, S. Chen et al. [80] and Xu et al. [77] engineered NIR-responsive hydrogels that generate ROS for melanoma eradication, albeit through distinct mechanisms. To prevent local recurrence and metastasis while accelerating the closure of large surgical defects, S. Chen et al. [80] designed an in situ sprayable calcium alginate matrix. This hydrogel encapsulates photosynthetic cyanobacteria alongside a targeted nanodrug featuring an ICG photosensitizer and an HA coating for CD44-selective cellular uptake. As illustrated in Figure 7, the system is applied to the postsurgical bed and activated via a dual-light irradiation sequence: a 635 nm red laser first stimulates photosynthetic oxygenation by the cyanobacteria to reverse the hypoxic microenvironment, followed by an 808 nm NIR laser to activate ICG and trigger phototoxicity. While this comprehensive approach demonstrated exceptional in vitro and in vivo efficacy—achieving complete tumor eradication without recurrence and accelerating neovascularized skin regeneration—its clinical translation may be hindered by the operational complexity of requiring sequential dual-laser irradiation [80].

Offering a simpler, single-nanomaterial alternative to the multicomponent oxygen-generating strategies discussed previously, Xu et al. [77] developed an injectable hydrogel for synergistic photothermal and oxidative therapy utilizing saffron-derived carbon dots (achieving a high photothermal conversion efficiency of 31.9%). While S. Chen et al. [80] addressed PDT oxygen limitations via active cyanobacterial photosynthesis, Xu et al. [77] relied on a distinct, secondary oxidative mechanism: near-infrared (808 nm) heating physically disrupts melanoma cell mitochondria, inducing electron leakage and a subsequent surge in intracellular ROS. Consequently, laser irradiation triggers pronounced cytotoxicity in B16F10 melanoma cells without requiring exogenous oxygen sources.

Moving beyond direct physical tumor ablation, HA-based hydrogels have also emerged as potent immunomodulatory platforms capable of reversing the immunosuppressive melanoma microenvironment, as highlighted by H. Chen et al. [98] and Hu et al. [71]. Specifically, H. Chen et al. [98] engineered an injectable HA hydrogel to overcome the inherent tendency of nanohydroxyapatite (nHAp) to aggregate and precipitate. In this system, surface HA acts as an active targeting ligand to stimulate cellular endocytosis. Following internalization, nHAp degrades within lysosomes, triggering a massive cytoplasmic calcium release that destabilizes mitochondria to induce apoptosis, while simultaneously accumulating in the endoplasmic reticulum to halt tumor cell protein synthesis. Crucially, this nHAp-loaded matrix drives the repolarization of tumor-associated macrophages from a pro-tumor M2 phenotype (CD206^+^) to an anti-tumor, pro-inflammatory M1 phenotype (iNOS^+^). By actively dismantling the immunosuppressive microenvironment, the platform achieved a melanoma growth inhibition rate of 78.6% [98]. However, whether this local immune reprogramming provides durable systemic protection against recurrence and metastasis, or merely represents a transient effect requiring repeated administration, remains to be established.

Advancing localized immunotherapy, Hu et al. [71] engineered an innovative, biocompatible HA hydrogel that functions as an in situ “immune cell factory” to prevent post-surgical melanoma recurrence. Implanted at the resection site, the hydrogel acts as a sustained reservoir for the local delivery of CAR-T cells (targeting the melanoma antigen CSPG4), IL-15 nanoparticles (to promote T-cell proliferation), and anti-PD-L1 antibody-conjugated platelets, which are activated by post-surgical inflammation to block immune-exhaustion pathways. Remarkably, this platform not only eradicated local residual tumors but also induced a robust abscopal effect, inhibiting the growth of distant, untreated tumors via the systemic recirculation of the locally primed CAR-T cells [71].

To overcome acquired drug resistance, Alfei et al. [69] developed an HA-based hydrogel for the topical treatment of metastatic cutaneous melanoma utilizing a novel triphenylphosphonium-based molecule termed BPPB. In this architecture, BPPB acts dually as the active chemotherapeutic agent and the network crosslinker, featuring highly pH-responsive release kinetics. The formulation relies entirely on non-covalent, physical crosslinking—a deliberate design choice that enables mild fabrication conditions and eliminates the need for potentially toxic chemical catalysts. Mechanistically, BPPB targets mitochondria to drive an extra-genomic mechanism of action that bypasses conventional drug resistance. Because mitochondria possess independent DNA distinct from the highly mutable nuclear DNA of cancer cells, their destruction triggers apoptosis via direct physicochemical damage rather than genomic pathways. Ultimately, this light-independent, ROS-mediated therapy demonstrated potent, dose- and time-dependent cytotoxicity with high selectivity against melanoma cells—specifically the MeOV and MeTRAV lines, which harbor distinct *BRAF* mutations—without risking the selection of resistant tumor clones [69].

### 7.3. Comparative Assessment of Efficacy, Safety and Translational Readiness

Taken together, while most studies on nanoparticle-functionalized CMC and HA hydrogels for melanoma demonstrate compelling proof-of-concept efficacy in vitro or in early-stage animal models, critical translational questions—such as long-term pharmacokinetics, systemic biodistribution, and manufacturing scalability—remain largely unaddressed. For instance, metal-oxide and metal-ion systems, such as the MnO_2_ nanoparticles employed by Enoch and Somasundaram [78], lack long-term in vivo toxicity evaluations, raising significant concerns given their known potential for off-target oxidative damage. As a precedent, cadmium-based quantum dots undergo core degradation that releases cytotoxic Cd^2+^ ions, generates membrane-damaging ROS, and exhibits size-dependent renal and splenic clearance, prompting regulatory phase-outs in other application domains [99]. Manganese-based systems warrant similar caution: recent repeated-dose toxicity data reveal that manganese oxide nanoparticles can induce measurable hepatic and renal histopathological alterations at doses as low as 100 mg/kg [100]. This reinforces the necessity for dose-ranging, long-term clearance studies before advancing manganese crosslinkers or fillers toward clinical hybrid platforms. Likewise, while immunomodulatory platforms—such as the macrophage-repolarizing nHAp hydrogel [98] and the CAR-T/platelet reservoir [71]—exhibit striking short-term efficacy, it remains unclear whether these effects are durable beyond the study window, or if their complex, cell-based manufacturing processes can be standardized for routine clinical application.

Table 1 summarizes the melanoma-specific systems discussed in Section 7.1 and Section 7.2 to facilitate direct comparisons of their efficacy, safety, and translational readiness. Two distinct asymmetries emerge from this analysis. First, the level of evidence differs markedly between the two polymers: all CMC-based systems remain at the in vitro stage (utilizing A375, MEL-39, or 3D bioprinted models) [25,63,76], with in vivo testing limited strictly to a chorioallantoic membrane irritation assay [25]. By contrast, seven of the eight HA-based systems were validated in animal models [5,71,77,80,96,97,98], leaving only the BPPB-loaded formulation restricted to cell culture [69]. Second, most in vivo studies relied on subcutaneous B16F10 tumors; only two [71,80] used post-resection models, which more closely reflect the clinical scenario targeted by post-surgical dressings.

Furthermore, reported efficacy data remain highly heterogeneous, complicating direct comparisons. Endpoints vary widely, encompassing tumor inhibition rates (e.g., 89.16% for the irradiated HA-His nanogel [96] and 78.6% for the nHAp hydrogel [98]), relative tumor volume reductions (~42% for the nanoceria system [97]), animal survival (80% at 42 days for the cyanobacteria-loaded hydrogel [80]), and cell viability reductions (~83% in 2D culture versus 56% in 3D models [76]). Moreover, these outcomes were achieved using disparate doses, irradiation parameters, initial tumor volumes, and follow-up periods. Rather than artificially ranking these systems, Table 1 makes this heterogeneity explicit and outlines the minimum parameter set that future studies on CMC–HA hybrids should report to enable standardized comparisons: nanoparticle type and dose, stimulus and irradiation conditions, tumor model and baseline volume, quantitative endpoints, and follow-up duration.

**Table 1:** Nanoparticle-functionalized and hybrid CMC- and HA-based systems evaluated against melanoma.

System	Polymer Role/Crosslinking	Nanoparticle/Active Agent	Therapeutic Modality (Stimulus)	Model (Validation Stage)	Main Outcome	Biosafety Evidence	Ref.
AgNP@CMC-DOX-CA hydrogel	CMC (DS 0.8/1.2): network, in situ reducing and capping agent, DOX carrier; citric acid crosslinking, mild pH and T	AgNP (in situ) + doxorubicin	Chemotherapy + Ag-mediated oxidative stress; antibacterial (pH-dependent release)	A375, HEK-293T control (in vitro, 2D); HET-CAM (in ovo)	Synergistic AgNP–DOX killing of A375; controlled release protects healthy cells; antibacterial in agar diffusion	MTT and crystal violet (HEK-293T); HET-CAM non-irritant	[25]
2-FA/CA/CUR@CMC-CA nanogel	CMC: anionic core with casein, solubilizes curcumin; self-assembly with citric acid + electrostatic layer-by-layer shell	Curcumin nanogel; folic acid/casein shell	Chemotherapy, folate-targeted; pH-dependent release (pH 6.8 vs. 7.4: 28 vs. 13% at 5 h; 54 vs. 30% at 12 h)	MEL-39 (in vitro); ex vivo skin permeation	IC50 13.1 vs. 15.8 (untargeted) and 18.3 µg/mL (free curcumin) at 48 h; higher apoptotic index	Blank nanogel non-cytotoxic; negligible hemolysis; CUR 3.47–4.15 µg/mL in skin layers	[63]
CMC–DOX prodrug hydrogels and patches	CMC: matrix and DOX-conjugating polymer; citric acid esterification (15% *m*/*m*)	None (DOX conjugate)	Chemotherapy with sustained, endocytosis-mediated delivery	A375 and HEK-293T (in vitro); MTT, 25 µM DOX, 6–48 h	Free DOX reduced viability to 35–40%, the hydrogels only to 65–75%; IC50 of the complexes 3.1–4.5× higher than free DOX	HEK-293T as normal-cell reference; confocal DOX uptake	[65,66]
CMC–fucoidan microneedle patch	CMC: dissolvable needle matrix; tips dissolved within 12 min (agarose skin model)	None (fucoidan as bioactive agent)	Antitumor activity of fucoidan; minimally invasive transdermal delivery	A375 in 2D and in a 3D bioprinted model (in vitro); ex vivo human skin (325–453 µm)	Viability reduced by ≈83% (2D) and ≈56% (3D) after 48 h	Assessed only in tumor models; no normal-skin panel	[76]
HA-His nanogel with DOX and Ce6	HA: nanogel backbone exposed for CD44 targeting; reversible Mn^2+^ coordination	Self-assembled Mn^2+^ nanogel; DOX + chlorin e6	Chemotherapy + PDT (laser), MRI-guided; responsive release	B16, C57 mice, s.c. ≈ 50 mm^3^, i.v. (in vivo)	Inhibition 89.2 ± 1.8% with irradiation vs. 57.3 ± 4.1% (free DOX + Ce6) and 42.2 ± 4.5% without Ce6; MRI confirmed accumulation	Stable body weight (15 days); H&E of five major organs	[96]
Gel-HCeC-CaO_2_ phototherapy hydrogel	HA only as ceria coating and CD44 ligand; matrix is a poloxamer F127/F68 thermogel (gelation ≈ 9 s at 37 °C)	HA-modified ceria linked to Ce6; CaO_2_	PDT with sustained in situ O_2_ generation (660 nm)	B16F10, athymic nude mice, s.c. ≈ 65 mm^3^, i.v. (in vivo)	Tumor volume reduced by ≈42%; HIF-1α decreased; no lung metastatic nodules	Stable body weight; H&E and TUNEL; biodistribution in five organs	[97]
CCa@TF/Ce6 hydrogel microneedle	HA grafted with 3-aminophenylboronic acid + PVA base (boro-ester bonds); PVA/PVP dissolvable tips	Carbon nanotubes@CaO_2_@tannic acid-Fe/Ce6	PTT + PDT + immunogenic cell death (NIR); self-supplied O_2_	B16F10, C57BL/6, s.c. ≈ 100 mm^3^ + skin regeneration (in vivo)	Tumor ablation followed by skin regeneration; lower tumor weight and Ki-67, higher TUNEL (no inhibition rate reported)	Body weight; H&E of major organs; hemolysis	[5]
HIL@Z/P/H sprayable hydrogel	HA coats the ZIF-8 nanodrug for CD44 uptake; matrix is calcium alginate, sprayed and gelled in situ	ICG and L-arginine in ZIF-8 (HIL@Z); cyanobacteria PCC 7942	PDT-generated ROS plus NO/RNS (808 nm) with photosynthetic oxygenation (635 nm)	Post-resection B16F10 model; lung metastasis (in vivo)	Tumor-cell survival 19.0% in vitro; animal survival 80% at 42 days; recurrence and metastasis inhibited; healing accelerated	Stable body weight (14 days); no toxicity in four organs	[80]
S-CDs@HA injectable hydrogel	Aldehyde-modified HA: injectable matrix, retention and release over 144 h; Schiff base with adipic dihydrazide	Saffron-derived carbon dots (≈2.4 nm)	PTT (808 nm; conversion efficiency 31.9%) with secondary ROS	B16F10, C57BL/6, s.c., intratumoral (in vivo)	Tumor volume increased only ≈50% by day 9 versus marked growth in controls; extensive necrosis	Hemolysis, body weight, organ histology; no systemic toxicity	[77]
nHAp-HA@HA injectable hydrogel	Thiolated HA: matrix and nanoparticle coating (dispersion, CD44); disulfide self-crosslinking	Hyaluronated nanohydroxyapatite	Ca^2+^ overload and ER stress; macrophage repolarization (M2→M1); microenvironment-responsive, no external stimulus	B16F10 mice, s.c. ≈ 50 mm^3^, 300 µg × 3 (in vivo)	Tumor inhibition 78.6%; Ki-67 and CD31 reduced; M1/M2 ratio increased	H&E of five major organs	[98]
CAR-T-P–aPDL1@gel	Acrylate-modified HA: implantable reservoir; UV photocrosslinking with crosslinker and photoinitiator	IL-15 nanoparticles; anti-PD-L1 platelets; CSPG4 CAR-T cells	Local immunotherapy; platelets activated by post-surgical inflammation	NSG mice, human WM115, resection cavity and distant tumor (in vivo)	Local recurrence and distant tumor growth inhibited; CAR-T cells detectable up to 4 weeks	No toxicity endpoints reported; CAR-T followed in blood	[71]
BPPB nanovesicles in HA hydrogel	HA matrix; BPPB acts as the non-covalent crosslinker (no chemical crosslinker or catalyst)	BPPB bola-amphiphile nanovesicles (triphenylphosphonium salt)	Mitochondria-targeted ROS, light-independent; pH-sensitive release	MeOV and MeTRAV, BRAF-mutant (in vitro)	IC50 49 nM on MeOV at 72 h; selectivity index up to 299 versus non-tumoral cells	Low cytotoxicity on non-tumoral lines; low hemolysis	[69]
Aminated HA/Bletilla striata polysaccharide hydrogel	Aminated HA as the network-forming polymer, combined with Bletilla striata polysaccharide	CuS@PDA@GOx (copper sulfide, polydopamine, glucose oxidase)	Starvation therapy (glucose consumption), CDT and PTT; continuous ROS cascade	Post-resection melanoma recurrence model in mice (in vivo)	Tumor recurrence inhibited, apoptosis increased and tumor vascularization reduced; immune repolarization, self-repair and prevention of bacterial infection	Not reported in the terms used here	[73]
GelS/HAD scaffold with PP/DOX nanomicelles	Dopamine-modified HA crosslinked with thiolated gelatin; porous, bioabsorbable, tissue-adhesive scaffold	Reduction-sensitive polymeric nanomicelles loaded with doxorubicin	Local post-surgical chemotherapy; physical capture of residual cells followed by RGD-mediated endocytosis; redox-responsive release	B16F10 post-resection model in mice (in vivo)	Dispersed tumor cells physically captured; postoperative recurrence reduced to 20%; lower expression of angiogenesis markers	Bioabsorbable; degraded gelatin fragments act as targeting ligands	[72]
Printable alginate/HA/gelatin bioink	HA provides CD44 binding within a printable alginate/gelatin network; ionic crosslinking	None	Not therapeutic: biofabrication of metastatic melanoma models for disease modeling and drug screening	In vitro constructs and rat arteriovenous loop model (in vivo)	Cell survival >80% and excellent shape fidelity; tumor growth, vascularization, intravasation, and lung metastasis reproduced	Not applicable (model system)	[75]

Values are as reported in the original studies and are not directly comparable, because doses, irradiation parameters, tumor volumes at treatment onset, and follow-up periods differ. In Refs. [80,97] HA is a nanoparticle coating rather than the hydrogel network; Refs. [65,66,75,76] contain no nanoparticles. Abbreviations: CUR, curcumin; DS, degree of substitution; ZIF-8, zeolitic imidazolate framework-8.

A final observation concerns the administration routes and the structural role of the polymers. Although broadly categorized as hydrogels for melanoma, several platforms are not applied locally to the tumor bed. Both the HA-His nanogel [96] and the nanoceria system [97] were administered intravenously; in the latter, the structural network is actually a poloxamer thermogel, with HA serving solely as a nanoparticle targeting ligand—a role it also plays in the calcium alginate matrix of the sprayable system [80]. Consequently, the number of studies applying a structural HA network directly to a melanoma wound bed is smaller than the literature initially suggests, underscoring the specific research gap addressed in this review. Regarding safety, available evidence is uniformly short-term, relying on body weight, hemolysis assays, and end-of-treatment histology [5,77,96,97,98], with no extended biodistribution data and a complete absence of toxicity endpoints for the CAR-T reservoir [71]. Ultimately, in terms of translational readiness, the simplest platforms—comprising a single nanomaterial dispersed within an injectable network [77,98]—are closest to scalable manufacturing, whereas the systems achieving the most robust responses rely on operationally complex dual-laser protocols with live bacteria [80] or highly customized, patient-specific cell engineering [71].

## 8. CMC–HA–Nanoparticle Hybrid Networks for Melanoma: A Design Framework

### 8.1. Existing CMC–HA Hybrid Networks in Biomedical Applications

To date, the literature lacks any reports detailing the development of HA-CMC hybrid networks specifically as nanoplatforms for melanoma treatment. Nevertheless, this polymer combination has been successfully harnessed for various other biomedical applications, including dermal fillers and aesthetic medicine [13,101], advanced wound healing [8,70,102], dura mater substitutes and anti-adhesion barriers in neurosurgery [103], and guided bone regeneration [104].

By manipulating the physicochemical properties of the hydrogel via network modifications and diverse gelation strategies, encompassing physical, chemical, and photo-induced approaches, these biomaterials can be precisely tailored to specific clinical requirements [18]. The chosen crosslinking strategy represents the fundamental mechanism governing the transition from a simple polymer blend to a structurally coherent three-dimensional matrix. Crosslinking agents drive this structural formation by establishing critical intermolecular interactions—whether via covalent bonds or non-covalent forces—thereby securing the architectural stability of the polymer network [18]. By strategically selecting these crosslinking mechanisms, researchers can carefully modulate essential properties such as matrix stiffness, elasticity, degradation kinetics, and biocompatibility, while enabling highly desirable clinical features like in situ injectability.

For instance, to formulate long-lasting dermal fillers, researchers frequently employ robust chemical crosslinkers like 1,4-butanediol diglycidyl ether (BDDE) to retard thermal and enzymatic degradation in vivo [13], or alternatively, utilize stable physical complexation with keratin to circumvent the need for synthetic chemical additives [101]. Conversely, for in situ applications, such as conformable wound dressings or neurosurgical interventions, visible- or UV-light-induced photocrosslinking utilizing photopolymerizable chemical groups is often preferred. This approach allows the precursor solution to be applied as a conformable liquid that rapidly solidifies to flawlessly replicate the geometry of irregular tissue defects [70,103]. Furthermore, for dynamic wound-healing applications, studies have utilized reversible crosslinking modalities (such as hemiacetal or dual-network crosslinking) that impart intrinsic self-healing properties and the mechanical flexibility required to accommodate the continuous movement of injured skin [8,102]. Table 2 summarizes these foundational studies exploring CMC-HA hybrid networks, detailing their respective biomedical applications and targeted crosslinking methodologies.

The studies summarized in Table 2 demonstrate the versatility of these hybrid hydrogels and confirm their synergistic advantages across non-oncological biomedical applications. Specifically, HA contributes CD44 receptor recognition, high water retention, and rapid soft-tissue regeneration—particularly in its low-molecular-weight formulations [70,104]. Conversely, CMC compensates for the inherently low thermal stability and mechanical strength of pure HA. This combination yields a robust polymeric network that withstands autoclave sterilization without compromising its physical or rheological integrity, while retaining the thickening, fluid-absorption, and moisture-retention capacities essential for clinical wound dressings [13,79,104,105].

Importantly, the biological influence of HA molecular weight is highly endpoint-specific and is not abolished by the crosslinking process. For instance, while low-molecular-weight HA actively promoted bone formation in guided-regeneration models [104], high-molecular-weight HA—but not its low-molecular-weight counterpart—significantly enhanced endothelial cell viability, tube formation, and scaffold vascularization following subcutaneous implantation. This angiogenic effect is largely attributed to CD44 receptor clustering, a biological mechanism that remains functionally intact even when high-molecular-weight HA is crosslinked into microporous annealed particle scaffolds [106].

**Table 2:** Key studies utilizing CMC–HA hybrid networks in the biomedical area.

Network Composition	Crosslinking Chemistry and Conditions	Biological Model	Mechanical Properties and Degradation	Main Outcome	Application	Ref.
Chondroitin sulfate (4% *w*/*v*), CMC (4% *w*/*v*) and HA (0.1% *w*/*v*) cryogel	Covalent etherification with BDDE in 0.2 M NaOH + 0.2 M KCl (pH 13); cryogelation at −17 °C	Adipose-derived stem cells; in vivo osteochondral defect, 12 weeks	Mechanics n.r.; degradation followed in PBS and in hyaluronidase (4000 U/mL), reported graphically	Sustained release of TGF-β1 and IGF-1; chondrogenic differentiation of stem cells; near-complete repair of the cartilage defect at 12 weeks	Articular cartilage tissue engineering	[107]
CMC (4% *w*/*v*), HA (20 mg/mL) and keratin	Stable physical complexation, without chemical crosslinkers	In vitro cytocompatibility assay	G′ 463 Pa; G″ 290 Pa; complex viscosity 117 Pa·s; swelling 95.87%; degradation n.r.	Restored skin volume and elasticity, with high stability and cell viability	Dermal/soft tissue filler	[101]
Methacrylated HA (HAMA) and methacrylated CMC (CMCMA), HA:CMC 1:3 to 3:1	In situ photo-crosslinking with LAP as photoinitiator under 400 nm visible light	L929 fibroblasts in vitro; rabbit dural defect model	Compressive elastic modulus +15.5% from HC13 to HC31; 35% degradation in 120 min in hyaluronidase (700 U/mL)	Watertight dural seal; barrier to scar-forming fibroblasts; prevented post-surgical adhesion; cell viability >80%	Dural substitute and anti-adhesion barrier	[103]
HA and CMC gel with micronized human acellular adipose matrix	Covalent crosslinking with BDDE; CMC added to HA at a 3:7 ratio, components and BDDE/NaOH solution heated to 50 °C, crosslinked at 50 °C for 3 h	Adipose-derived stem cells; in vivo adipose tissue formation	Storage and loss moduli and complex viscosity measured against a commercial HA filler (25 °C, 0.1–10 Hz, 1.0% strain); enzymatic degradation in hyaluronidase at 40 °C over 48 h, reported graphically as retention rate	Preserved adipose growth factors; 84% adipogenic differentiation in 7 days; formation of new adipose tissue in vivo	Soft tissue augmentation/dermal filler	[105]
Low-molecular-weight HA (200 kDa) and CMC	Physical blending; HA depolymerized by γ-irradiation (1 kGy/h, 20 h, 22 °C) before mixing	Dental pulp stem cells; rabbit calvarial defects	n.r.	Stem cell proliferation through CD44 and MAPK signaling; about twice the new bone formation obtained with high-molecular-weight HA/CMC	Guided bone regeneration	[104]
HA and dialdehyde CMC (DCMC 0.5% *w*/*v*) loaded with gallic acid (0.02% *w*/*v*)	Freeze–thaw gelation with dynamic hemiacetal interactions (no synthetic crosslinker)	L929 fibroblasts (viability > 70%); rat full-thickness wound model	Compressive strength 86.7 kPa (HA/DCMC) and ≈45.65 kPa for the gallic acid-loaded gel; degradation n.r.	Wound closure of 76.29 ± 4.08% at day 10 versus 33.62 ± 6.24% in the control; regeneration of hair follicles and sweat glands; antibacterial activity and radical scavenging of 70.24 ± 2.8%	Skin wound management	[102]
HA-catechol (0.5–1% *w*/*v*) and CMC-methacrylate (0.5–1% *w*/*v*) with PEGDA and MBA, loaded with rhFGF2	In situ photo-crosslinking under 365 nm UV light with a photoinitiator	Fibroblasts in vitro (viability > 70%); in vivo wound model	Compression testing performed (elastic modulus not assessed); erosion below 10%	100% in vitro wound closure within 36 h; healing accelerated by about 30% in vivo; sustained delivery of rhFGF2	Active wound dressing for chronic wounds	[70]
Oxidized HA, modified CMC (HCMC 1% *w*/*v*) and PVA (6% *w*/*v*) loaded with sodium bicarbonate (1.25–5%)	Double crosslinking: dynamic hydrazone/Schiff base bonds plus physical crystallization of PVA (freeze–thaw)	L929 fibroblasts; rat full-thickness skin defects (8 mm)	Adhesion strength from 8.43 kPa (control) to 14.54 kPa at 5% NaHCO_3_; ≈80% mass loss within 14 days, tunable with the NaHCO_3_ content	Accelerated wound closure, epidermal regeneration, reduced inflammation and increased collagen deposition; about 90% killing of *S. aureus* and higher activity against *E. coli*	Infected skin wounds (antibiotic-free)	[8]
HA and CMC composite	Covalent etherification with BDDE under alkaline conditions	In vitro cytocompatibility assay	Highest viscoelastic moduli at an HA/CMC ratio of 1:1, largely retained after autoclaving at 121 °C for 20 min (measured at 20 and 37 °C); degradation n.r.	Overcame the thermal instability and low mechanical strength of HA alone; withstood autoclave sterilization without loss of properties	Soft tissue/dermal filler	[13]

Abbreviations: CMCMA, methacrylated CMC; DCMC, dialdehyde CMC; HCMC, modified CMC; IGF-1, insulin-like growth factor 1; LAP, lithium phenyl-2,4,6-trimethylbenzoylphosphinate; MBA, N,N′-methylenebisacrylamide; PEGDA, poly(ethylene glycol) diacrylate; PVA, poly(vinyl alcohol); rhFGF2, recombinant human fibroblast growth factor 2; TGF-β1, transforming growth factor β1.

### 8.2. Crosslinking Selection for a Melanoma-Targeted CMC–HA Hybrid Hydrogel

Translating the complementary relationship of CMC, HA, and nanoparticles into a functional hybrid network requires the strategic selection of a crosslinking architecture. Ultimately, this choice dictates whether the CMC–HA–nanoparticle platform behaves as a mechanically robust, long-term implant or a rapidly degrading, readily injectable system [108]. Drawing upon the crosslinking mechanisms discussed in Section 8.1 and their outcomes (Table 2), a dual-network design emerges as a highly promising theoretical approach for a melanoma-targeted CMC–HA hybrid. In this design, the CMC backbone is chemically crosslinked to provide the primary mechanical scaffold and the sterilization resistance necessary for a post-surgical dressing. This structural foundation can be achieved via citric acid-mediated esterification or EDC/NHS-mediated amidation. In the latter, EDC activates CMC carboxyl groups to form a reactive O-acylisourea intermediate, which NHS stabilizes into a reactive ester that bonds with a primary amine, which must be added to the system by a third molecule, to establish the covalent network [18].

Complementing a robust CMC scaffold, a secondary HA sub-network can be formed in situ under physiological conditions. This HA network could be covalent yet cytocompatible (e.g., via thiol–(meth)acrylate Michael addition yielding thioether bonds [54]), dynamic-covalent (hydrazone bonds), or purely physical (polyelectrolyte complexation or metal–ligand coordination [55,85]). Crucially, these mild in situ methods confer injectability, rapid gelation at body temperature, and stimuli-responsive drug release without compromising HA’s receptor-mediated targeting. Because the carboxylate groups on HA are essential for both CD44 recognition and nanoparticle capping, any chemical modification at these sites proportionally reduces the fraction available for active targeting.

This competition for carboxylate groups necessitates a careful selection of crosslinking chemistries based on their specific consumption profiles. Approaches such as esterification with citric acid or the photocrosslinking of methacrylated polysaccharides react exclusively through the polymer’s hydroxyl groups. Consequently, they leave the carboxylate domains intact, preserving both CD44-binding capacity and nanoparticle-capping sites. Purely ionic or physical interactions offer similar benefits by avoiding covalent bonds entirely. While etherification with BDDE also targets hydroxyls, its requisite strongly alkaline conditions are generally incompatible with pre-formed nanoparticles and sensitive therapeutic payloads; thus, while carboxylates are preserved, overall payload capacity is severely restricted.

At the opposite extreme, carbodiimide (EDC/NHS) amidation directly consumes the precise carboxylates required for CD44 recognition and nanoparticle stabilization. For this reason, we propose restricting this specific chemistry entirely to the structural CMC backbone. Quantitatively, HA modification degrees exceeding ~40% significantly interfere with CD44 binding [106,109], establishing a strict upper limit for functionalization. To optimally preserve active targeting and receptor-mediated signaling, biomaterial designs must aim for much lower modification levels (e.g., ~10% to 15%). Intermediate approaches include periodate oxidation (which opens sugar rings to generate aldehydes for dynamic networks) and targeted carboxylate thiolation (the precursor to thiol-Michael and disulfide crosslinking). In these cases, a low degree of modification suffices for gelation while preserving the majority of binding sites; notably, in thiolated systems, the modified polymer inherently aids in dispersing and targeting the nanoparticles [98]. Conversely, grafting catechol groups onto HA [70] consumes carboxylates and requires careful evaluation against this same targeting trade-off. Therefore, a CMC–HA hybrid engineered for active CD44 targeting should concentrate its covalent crosslinking on the CMC network, keeping HA modification strictly to the minimum required for gelation.

Table 3 compares the crosslinking chemistries available for CMC–HA networks across several key parameters: synthesis conditions, injectability, mechanical strength, degradation kinetics, stimuli-responsiveness, and their specific impact on the carboxylates critical for CD44 recognition and nanoparticle capping. Furthermore, it outlines sterilization constraints, regulatory considerations, and the proposed structural role for each chemistry within the hybrid platform.

### 8.3. Nanoparticle Selection

Regarding nanoparticle selection, the safety profiles discussed throughout Section 7.1 and Section 7.2 clearly favor gold nanoparticles/nanorods, carbon-based dots, and iron- or cerium-oxide nanostructures as the most viable initial candidates for a hybrid CMC–HA platform. These specific nanomaterials successfully integrate photothermal, photodynamic, or oxygen-generating functionalities with comparatively well-characterized in vivo clearance behaviors. Consequently, they represent a far more realistic translational route than cadmium-based quantum dots or high-dose manganese oxide, which suffer from severe toxicity and translational limitations (as detailed in Section 7.3) [99,100].

For hypoxia management specifically, the choice between single- and multi-component oxygen-generating strategies also affects hybrid gel stability [110,111]. A single-component source such as calcium peroxide offers mechanistic simplicity but generates H_2_O_2_/O_2_ via an uncontrolled, burst-type hydrolysis reaction, producing alkaline byproducts that can shift the local microenvironment by several pH units [112]; this pH shift could interfere with ionic and hydrogen-bonding interactions that stabilize a CMC-HA network. Multi-component oxygen generators that pair a peroxide source with a catalytic or enzymatic regulator—for example, catalase supplementation or a protective polymeric barrier that limits water access to the peroxide core—have already been shown to reduce this burst effect and sustain more stable, titratable O_2_ release in other hydrogel systems [112,113], which may better preserve hybrid gel integrity over the days-to-weeks timescale required for a post-surgical wound bed. Nonetheless, this specific trade-off has not yet been tested in a CMC-HA hybrid and represents a concrete open question for future formulation studies.

Table 4 systematically compares the diverse classes of nanoparticles utilized across the reviewed systems. This comparison evaluates their primary theranostic functions, reported experimental performance, imaging capabilities, biodegradation pathways, clearance mechanisms, and primary toxicity concerns. Furthermore, it outlines their current stage of development and proposes a clear prioritization framework for integrating these specific nanoparticle classes into a CMC–HA platform targeted for melanoma therapy.

## 9. Translational and Regulatory Considerations

Beyond materials design, translating a multi-component CMC–HA–nanoparticle platform requires navigating a regulatory pathway significantly more complex than those established for single-polymer hydrogels. Because these systems integrate a biopolymer matrix, inorganic or metal-based nanoparticles, and often an active chemotherapeutic or photosensitizing payload, they typically classify as combination products. Consequently, they demand a coordinated regulatory review of their device, drug, and potentially biologic constituents, precluding a straightforward single-pathway approval [118,119].

The specific regulatory jurisdiction cannot be inferred merely from the dosage form. Instead, it is dictated by the platform’s composition, intended use, and—most critically—its primary mode of action (PMOA). Regulators establish this determination through formal procedures rather than leaving it to developer presumption. In the United States, combination product definitions and lead-center assignments are governed by 21 CFR Part 3. If the PMOA is ambiguous, developers must submit a Request for Designation to the Office of Combination Products [120]. Therefore, several FDA pathways are possible: a device-led review, where a novel dressing lacking a predicate proceeds via Premarket Approval or the De Novo route, or a drug-led review, where the payload provides the principal therapeutic action.

In the European Union, classification follows Annex VIII of Regulation (EU) 2017/745. Devices incorporating nanomaterials are classified based on their potential for internal exposure. A device incorporating an ancillary medicinal substance automatically falls into the highest risk class. Conversely, if the principal intended action is pharmacological, immunological, or metabolic, the system is regulated as a medicinal product, requiring the device component to be assessed by a notified body under Article 117 [121]. These frameworks are presented as potential scenarios rather than a determined route; for conceptual platforms, the definitive pathway requires early engagement with competent authorities. Ultimately, these regulatory realities are highlighted because they must directly inform the initial design choices discussed in Section 8.

For the nanoparticle components specifically, regulatory bodies increasingly demand long-term biodistribution, clearance, and degradation data that extend far beyond the short-term in vitro and early in vivo endpoints typical of current literature. This requirement is particularly stringent for non-biodegradable or heavy-metal-based nanomaterials [99,119]. Addressing this translational gap early in the design phase—for example, by prioritizing nanoparticles with established clearance pathways alongside biopolymer crosslinkers already utilized in approved devices—will substantially bridge the gap between current proof-of-concept platforms and clinically viable CMC–HA nanotheranostics.

Finally, advancing these hydrogels toward clinical translation necessitates comprehensive biological evaluation in strict accordance with the ISO 10993 series for medical devices, including testing for cytotoxicity, irritation, sensitization, and hemocompatibility [122]. Future studies must systematically establish critical translational parameters, including the compatibility of the chosen crosslinking chemistry with the nanoparticle formulation, the hydrogel’s resilience to standard sterilization protocols, and its long-term storage stability [123,124].

As summarized in Figure 8, conventional melanoma management remains hindered by factors such as systemic toxicity, incomplete tumor clearance, and the risk of secondary infection, particularly within the physiologically complex skin microenvironment. The proposed hybrid hydrogel addresses these limitations synergistically: CMC provides structural integrity and fluid-handling capacity, while HA mimics the extracellular matrix and enables CD44-mediated biological recognition. Crucially, the core innovation of this platform lies in its integration with functional hybrid nanoparticles. This combination allows for the seamless consolidation of diagnostics and multiple therapeutic modalities into a single wound-dressing platform, offering a multifunctional alternative for localized melanoma management and forming the foundational strategy proposed in this article.

## 10. Conclusions and Future Aspects

This review surveys recent advances in the development of hybrid CMC–HA hydrogels for melanoma applications, highlighting the challenges and remaining gaps in transitioning these biomaterials into effective nanostructured systems. While combining carboxymethyl cellulose and hyaluronic acid into a single network synergistically merges mechanical robustness with targeted cell biology, the true potential of these platforms lies in their integration with hybrid nanoparticles as the core innovation. Although individual nanoparticles (e.g., QDs, AgNPs, and AuNPs) show promise in treating skin diseases, hybrid networks integrating both these specific polysaccharides and functional nanoparticles for melanoma management remain unexplored, representing a critical gap in the current literature.

Future studies should prioritize nanoparticle systems with proven safety and biocompatibility profiles over heavy-metal-based alternatives, which are limited by toxicity and a lack of data regarding long-term biological interactions. Future work must also evaluate which cross-linking strategies (e.g., physical, chemical, photo-induced, or hybrid) best balance mechanical stability with the injectability and rapid gelation required for post-surgical melanoma wound beds. Given the broad design space of these platforms, systematic studies are essential to optimize critical formulation parameters—such as the polymer-to-crosslinker ratio, nanoparticle type, and surface functionalization—to achieve the desired biological performance.

It is also important to note that most of the individual CMC- and HA-based systems reviewed here remain limited to in vitro validation. Advancing hybrid CMC–HA nanoplatforms will require robust in vivo studies to substantiate their translational potential for human skin, which possesses unique physiological characteristics. Specifically, detailed studies are needed to determine biodistribution, long-term safety, and therapeutic efficacy in highly representative melanoma models.

Ultimately, this review aims to guide future research on hybrid hydrogel–nanoparticle architectures as bifunctional post-surgical dressings. By eliminating residual melanoma cells through combined nanotherapies while simultaneously promoting tissue repair and preventing opportunistic infections, these platforms pave the way for multifunctional, clinically relevant approaches to melanoma treatment.

## Figures and Tables

**Figure 1:**
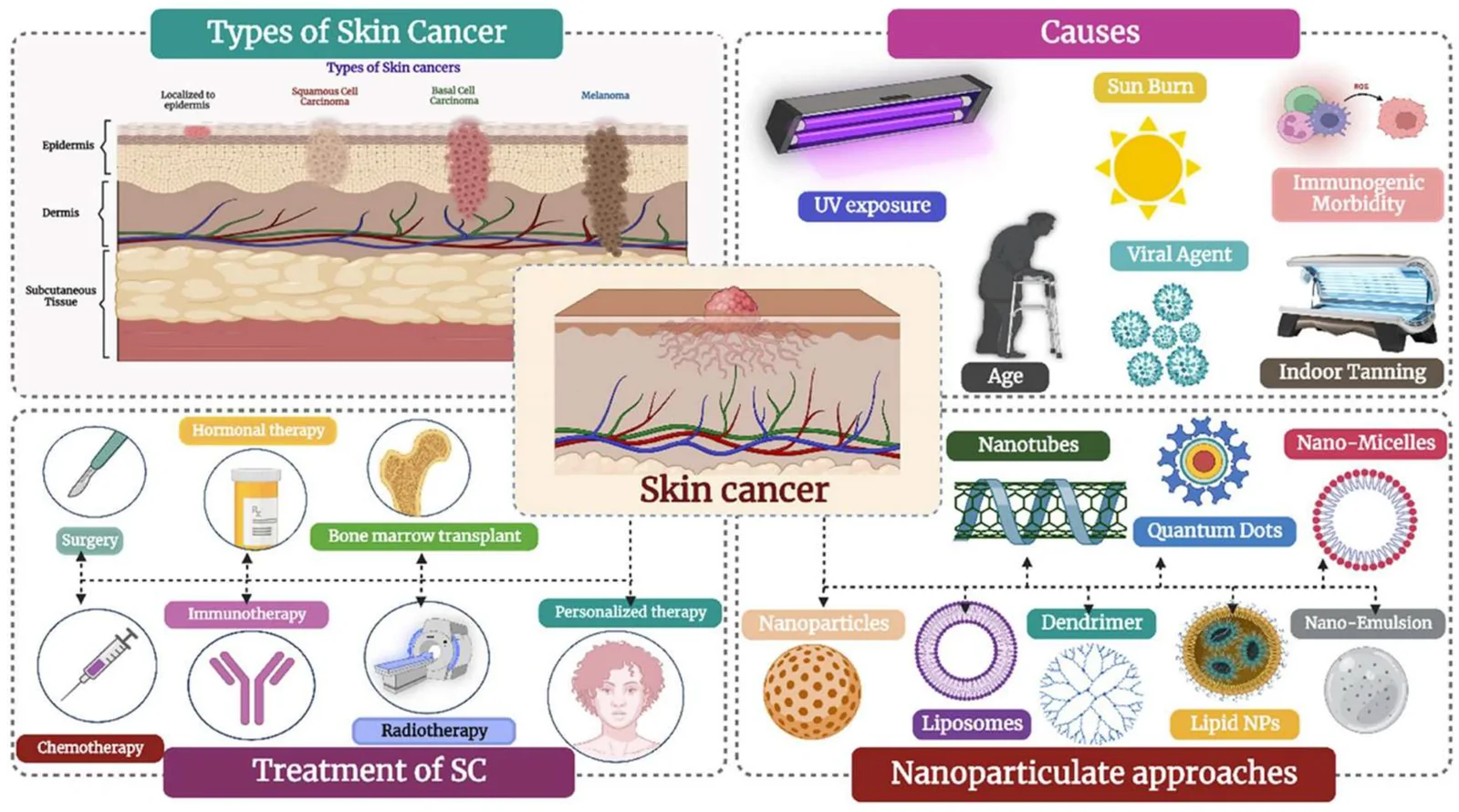
Types and causes of skin cancer with the main treatment and nanoparticle approaches. Reprinted with permission of [1] under the terms of the Creative Commons Attribution (CC BY 4.0) license.

**Figure 2:**
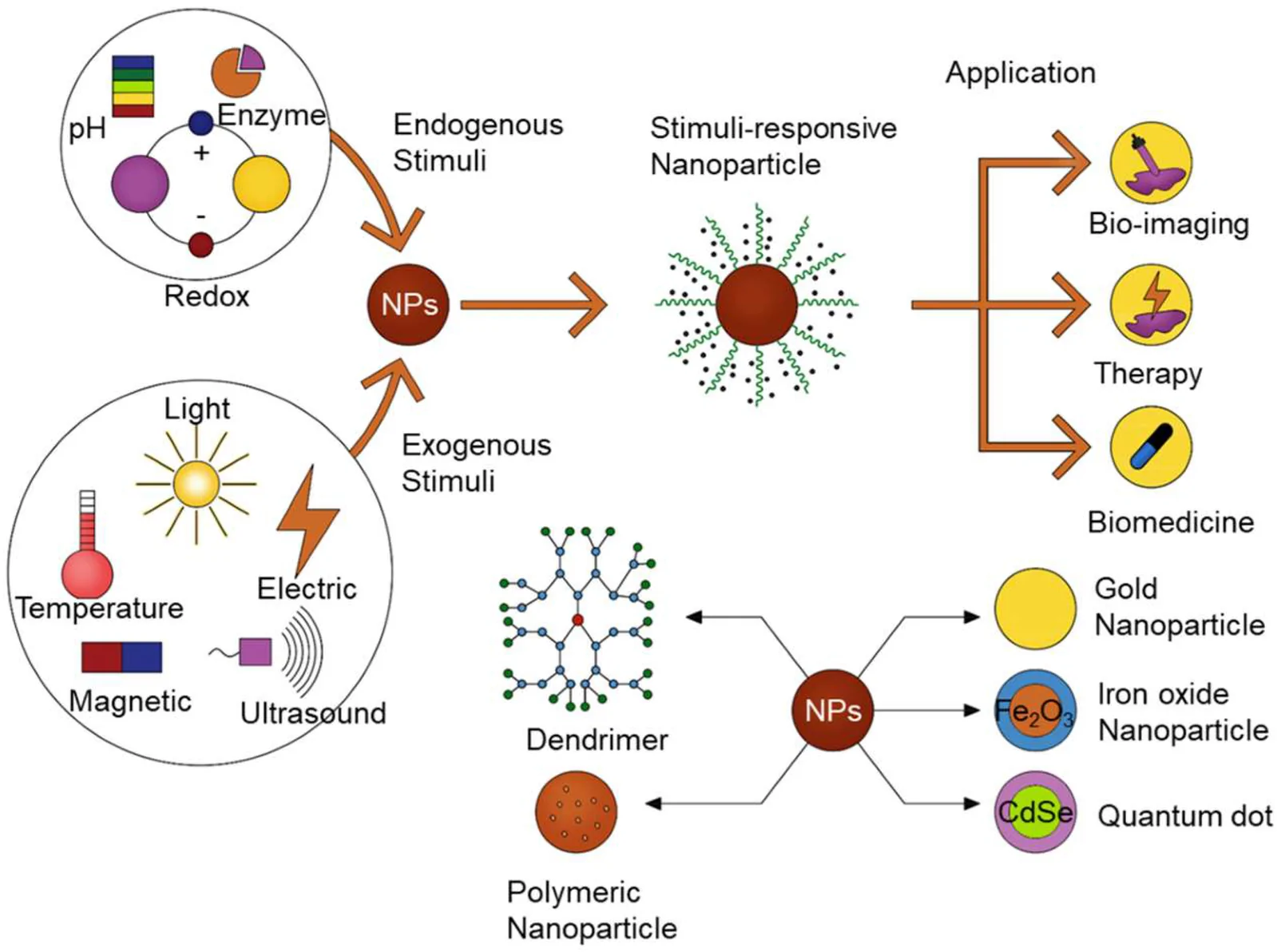
External and internal stimuli in nanotherapeutics for cancer theranostics. Reprinted with permission of [38] under the terms of the Creative Commons Attribution (CC BY 4.0) license.

**Figure 3:**
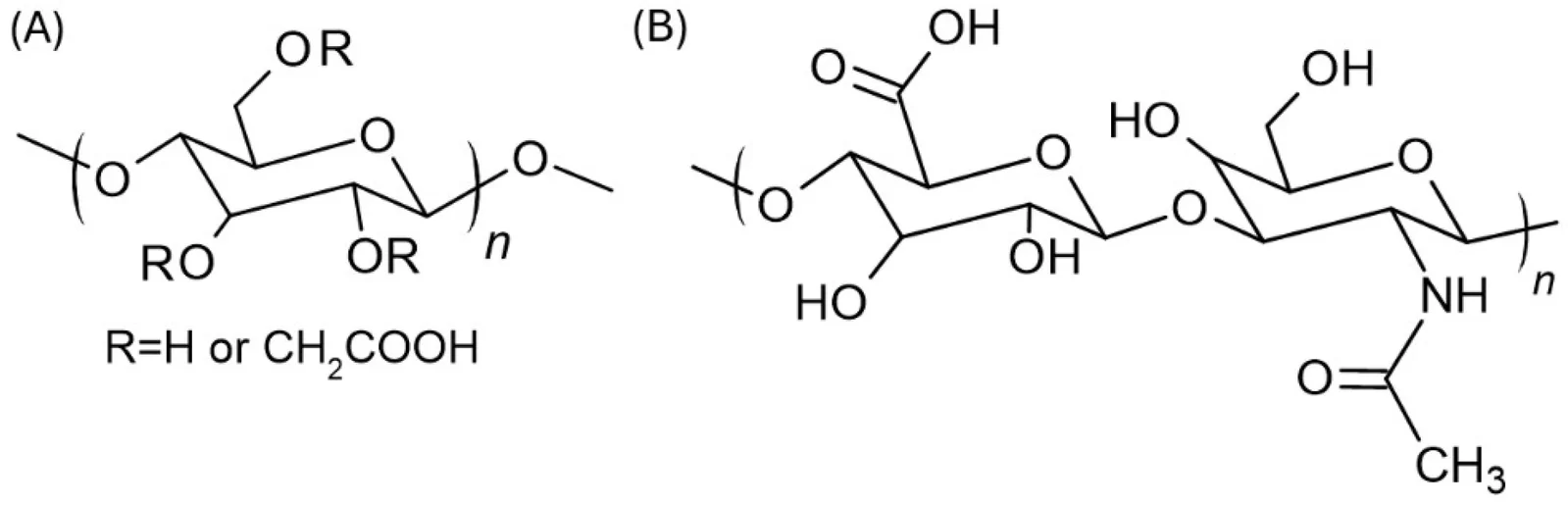
Chemical structure of (**A**) CMC and (**B**) Hyaluronic Acid. Adapted and reprinted with permission [13] under the terms of the Creative Commons Attribution (CC BY 4.0) license.

**Figure 4:**
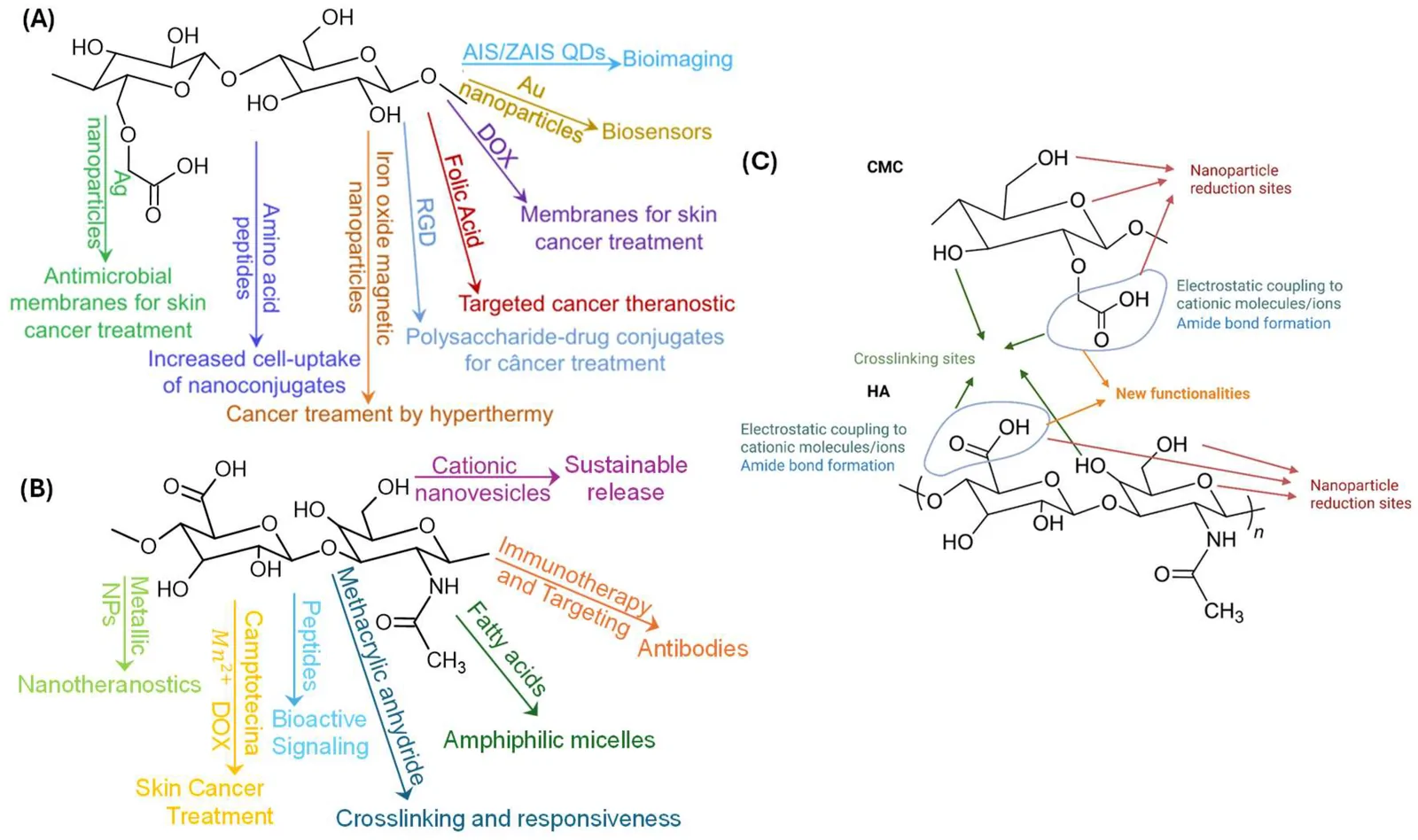
(**A**) Diverse bioapplications of carboxymethylcellulose (CMC), exploiting the reactivity of its hydroxyl and carboxyl groups toward conjugation with: Ag nanoparticles for antimicrobial mem-branes [25]; amino acid peptides for increased cellular uptake of nanoconjugates [60]; iron oxide magnetic nanoparticles for hyperthermia-based cancer treatment [61]; RGD for polysaccharide-ride-drug conjugates [62]; folic acid for targeted cancer theranostics [63]; AIS/ZAIS quantum dots for bioimaging [60,64]; Au nanoparticles for biosensing [14]; and doxorubicin (DOX) for antitumor membranes for skin cancer treatment [65,66]. (**B**) Bioconjugation strategies for hyaluronic acid (HA), exploiting the reactivity of its functional groups toward: deacetylation for exposure of free active amino sites [55]; conjugation with metallic nanoparticles for nanotheranostics [33]; DOX for skin cancer treatment [67]; RGD peptides for bioactive signaling [62]; fatty acid conjugation for amphiphilic micelle self-assembly [68]; cationic nanovesicles for sustained release [69]; camptothecin for skin cancer treatment [67]; methacrylic anhydride for photocrosslinkable properties [70]; and antibodies for immunotherapy and targeting [71]. (**C**) A highlight of potential modification sites of a CMC and HA repeating units with different aims (Created in BioRender. Miranda, D. (2026) https://BioRender.com/sszbyfe (accessed on 2 September 2026).

**Figure 5:**
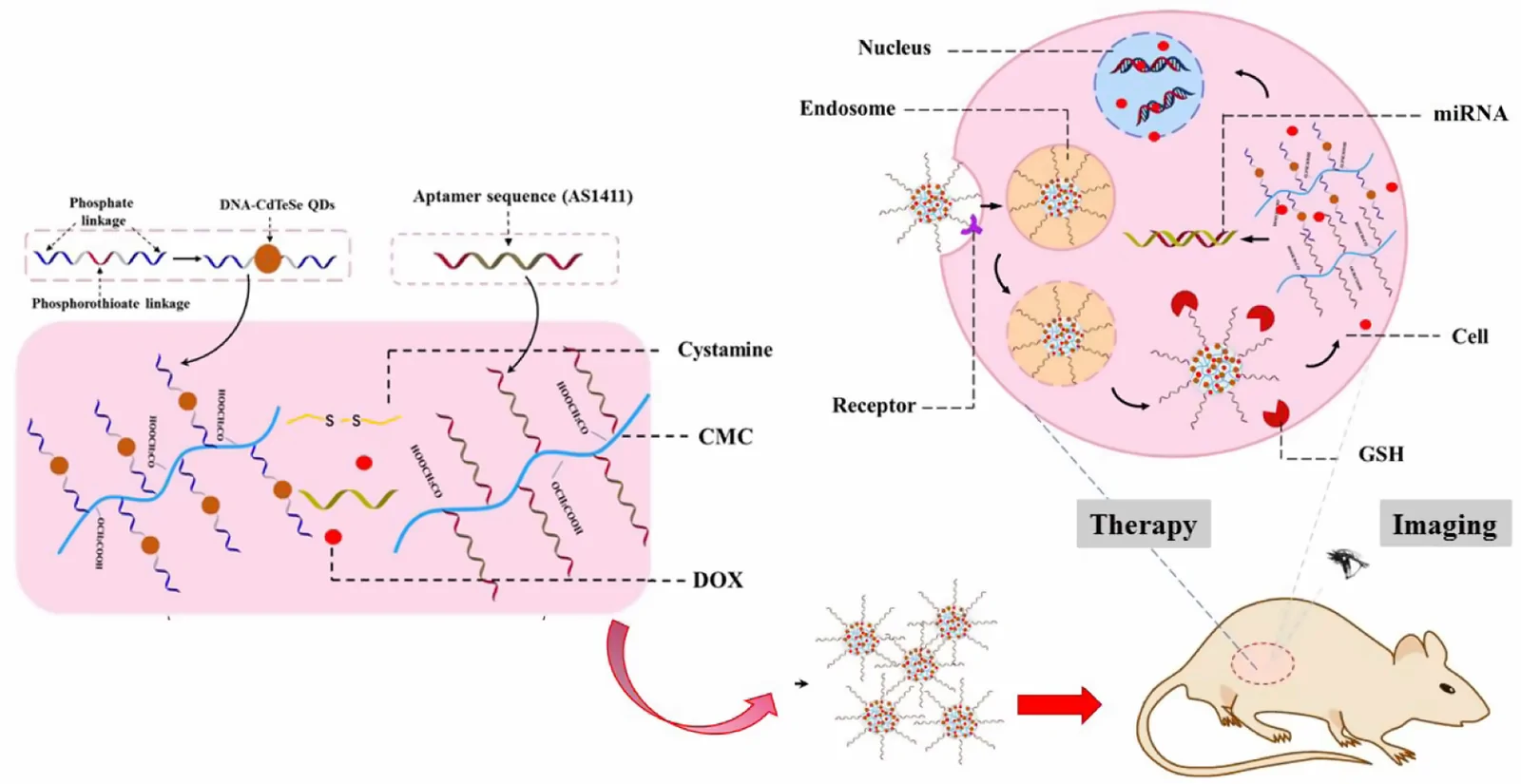
Multifunctional carboxymethylcellulose (CMC) nanohydrogel structured with DNA-templated CdTeSe quantum dots (QDs) for tumor theranostics, combining fluorescence imaging with dual-stimulus (GSH/miRNA) triggered doxorubicin release. Reprinted with permission of [64] under the terms of the Creative Commons Attribution-NonCommercial (CC BY-NC 3.0) license.

**Figure 6:**
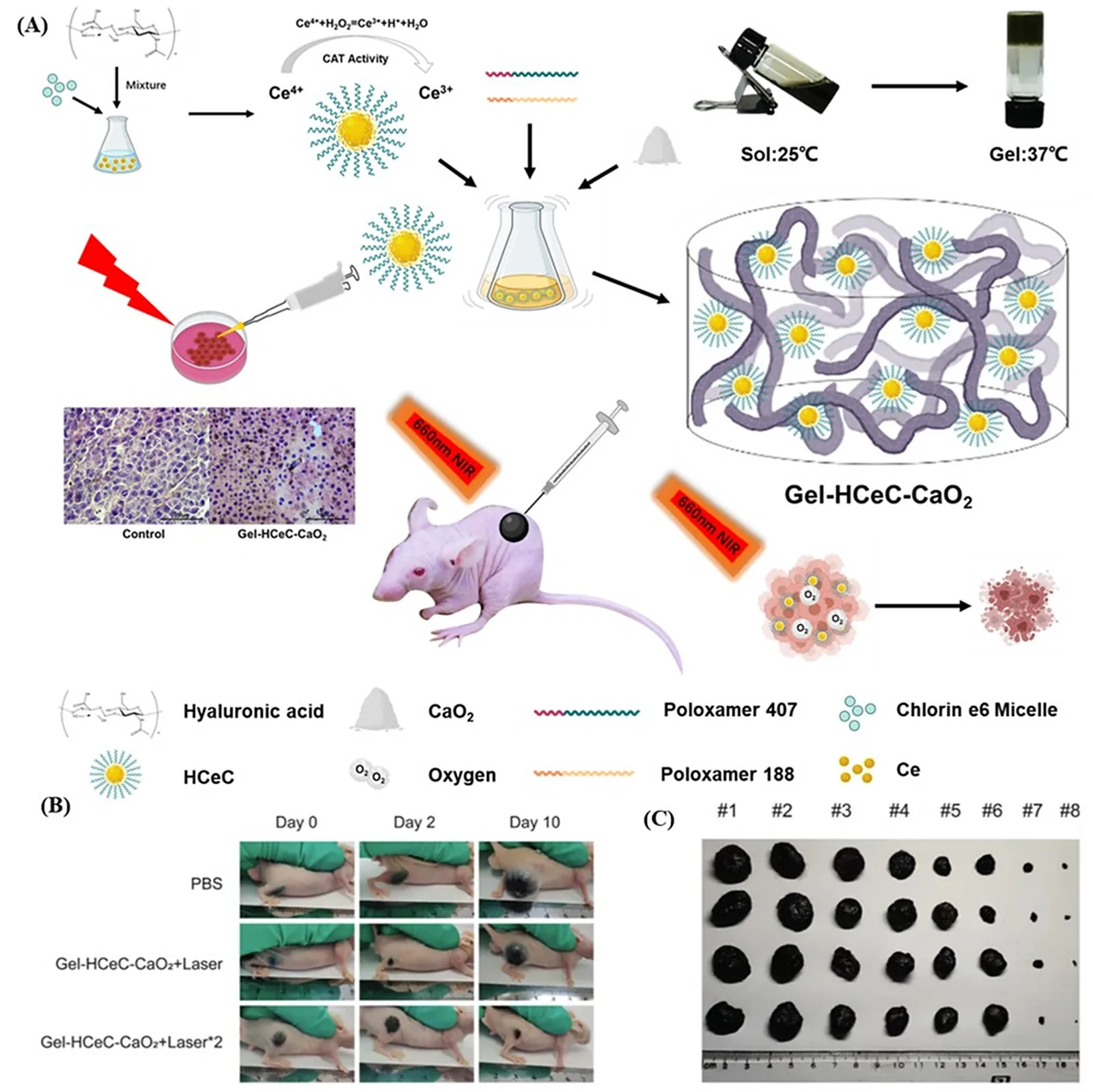
(**A**) Hyaluronic acid (HA)-modified cerium oxide (ceria) nanoparticles covalently linked to the photosensitizer chlorin e6 (Ce6), incorporated into an injectable HA hydrogel for sustained oxygen-generating phototherapy of melanoma. (**B**,**C**) The tumors of mice in each group were photographed (#1: PBS; #2: Gel-CaO_2_; #3: HCeC; #4: Gel-HCeC-CaO_2_; #5: HCeC+Laser; #6: Gel-HCeC-CaO_2_+Laser; #7: HCeC+Laser*2; #8: Gel-HCeC-CaO_2_+Laser*2). Reprinted with permission of [97], distributed under the terms of the Creative Commons Attribution (CC BY 4.0) license.

**Figure 7:**
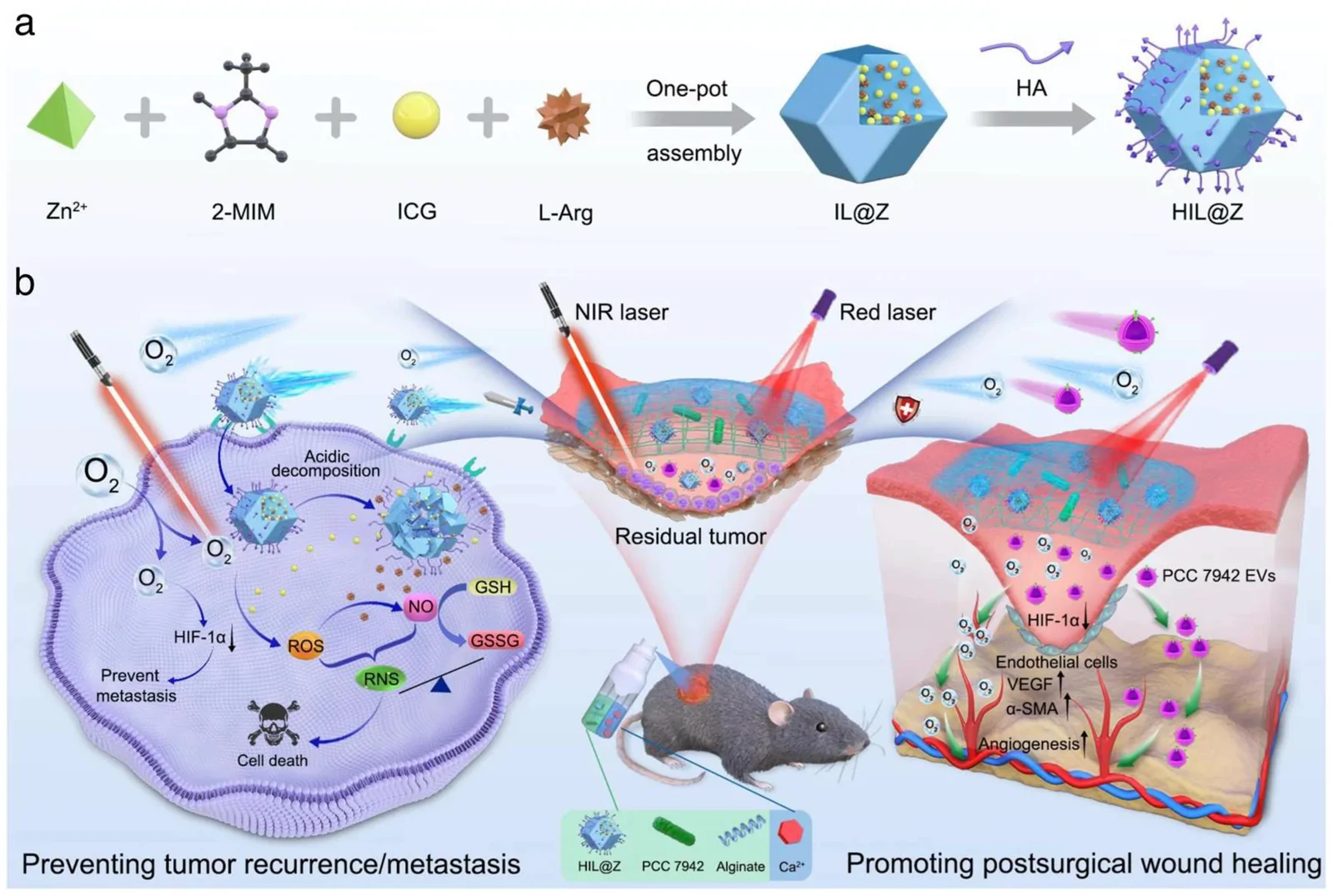
Schematic illustration of the sprayable HIL@Z/P/H therapeutic hydrogel encapsulating a tumor-targeted nanodrug (HIL@Z) and photosynthetic cyanobacteria (PCC 7942) for postsurgical melanoma treatment. (**a**) Preparation of the HIL@Z nanodrug. (**b**) In situ formation and mechanism of action of the sprayed hydrogel: photosynthetically generated O2 relieves the hypoxic tumor microenvironment and fuels intracellular cascade reactions (ROS, NO, RNS) that prevent local recurrence and distant metastasis of residual tumor cells, while the sustained O2 supply and PCC 7942-secreted extracellular vesicles jointly promote angiogenesis and accelerate wound healing. Reprinted with permission of [80], distributed under the terms of the Creative Commons Attribution (CC BY 4.0) license.

**Figure 8:**
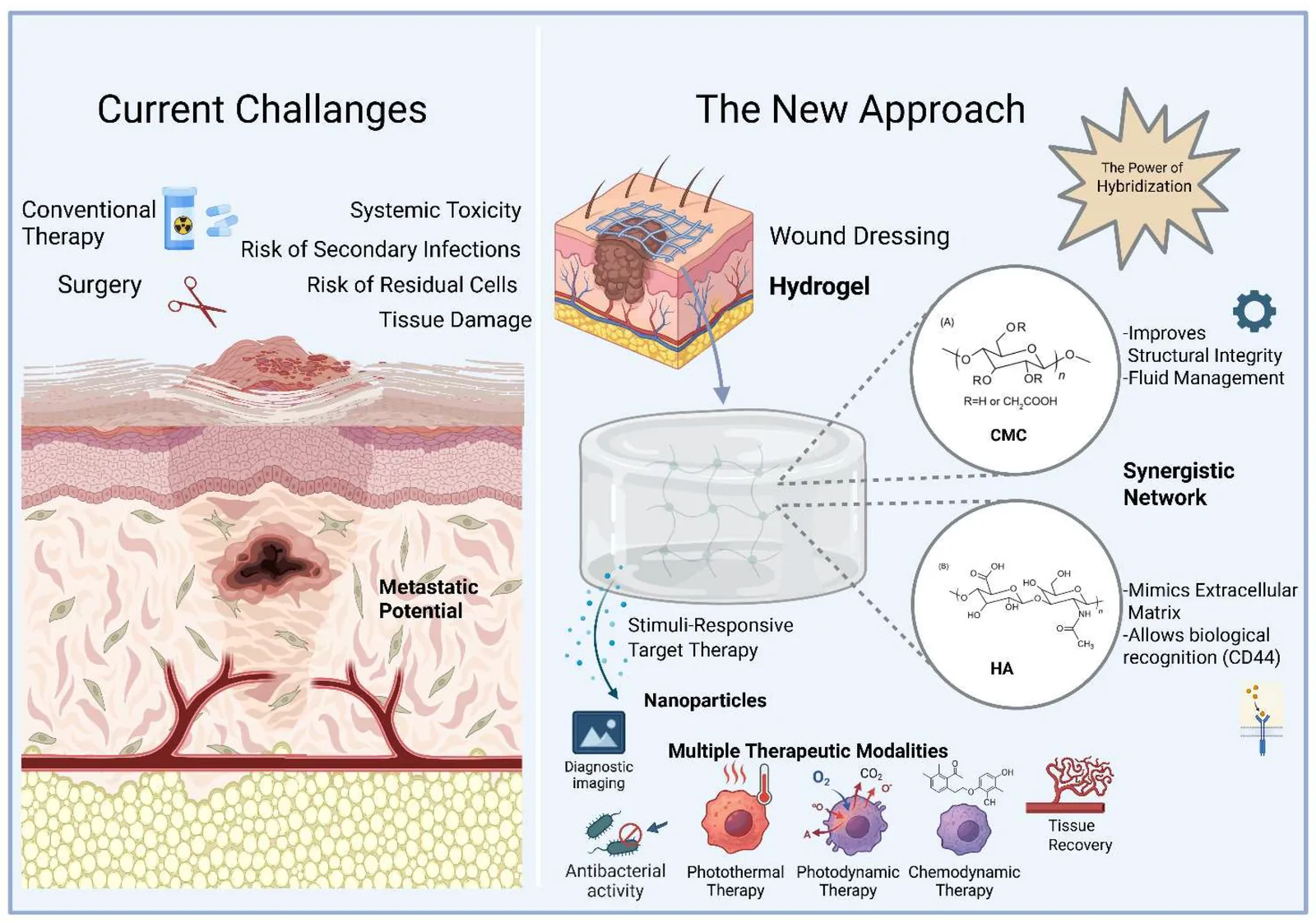
Graphical summary of the current challenges in melanoma skin cancer management (systemic toxicity, risk of secondary infection, residual tumor cells, tissue damage and metastatic potential associated with conventional surgery and therapy) and of the CMC/HA hybrid hydrogel-based approach discussed throughout this section. The synergistic combination of CMC (improved structural integrity and fluid management) and HA (extracellular-matrix mimicry and CD44-mediated biological recognition) forms a wound-dressing hydrogel network capable of stimuli-responsive, nanoparticle-mediated multiple therapeutic modalities—including diagnostic imaging, antibacterial activity, photothermal, photodynamic, and chemodynamic therapy—to support tissue recovery. Created in BioRender. Miranda, D. (2026) https://BioRender.com/23ywg85 (accessed on 2 September 2026).

**Table 3:** Decision matrix linking crosslinking chemistry to the requirements of a melanoma-targeted CMC–HA platform.

Crosslinking Chemistry	Typical Conditions	Injectability, Mechanical Strength and Degradation	Stimuli-Responsiveness	Sterilization and Regulatory Notes	Suggested Role in a Melanoma-targeted CMC–HA Hybrid	Ref.
Esterification with citric acid	Citric acid 15% (*m*/*m* of polymer), mild pH, thermal curing of the cast film	Not injectable: films and patches are pre-formed; high strength for a dressing; slow degradation by ester hydrolysis	pH-dependent swelling and release	Food-grade, non-toxic crosslinker; compatible with dry storage; thermolabile drugs must be loaded after curing	CMC backbone—first choice for the mechanically robust, sterilizable dressing layer	[18,25,65,66]
Etherification with BDDE	0.2 M NaOH (pH ≈ 13) or NaOH solution at 40–50 °C; cryogelation at −17 °C in the cryogel route	Not injectable as formed; highest strength of the chemistries reviewed, withstands autoclaving in HA/CMC composites; very slow degradation (months)	Minimal	Used in approved dermal fillers; residual BDDE must be controlled	Not recommended for a nanoparticle-loaded hybrid; retain only for a pre-formed, filler-type component	[13,52,105,107]
Carbodiimide (EDC/NHS) amidation	Aqueous buffer at pH ≈ 4.5–6, room temperature; EDC is released as a water-soluble urea and washed out	Usually pre-formed; moderate to high strength, tunable with the bridge length; moderate degradation, governed by backbone hydrolysis	Low, unless a cleavable bridge such as cystamine is used	Zero-length crosslinker, no residue incorporated; requires purification	Use sparingly and preferentially on CMC, to graft functional groups; avoid on HA so that CD44 sites are preserved	[18,70,85]
Dynamic hydrazone, imine (Schiff base) and hemiacetal	Aqueous, room temperature, gelation in seconds to minutes; hemiacetal networks formed by freeze–thaw cycles	Injectable, sprayable, and self-healing; low to moderate strength, improved by a second network; fast degradation (days to weeks), accelerated at acidic pH	High: acid-labile bonds release the payload in the acidic tumor microenvironment	No toxic crosslinker required; residual aldehydes must be controlled	HA sub-network—first choice for injectability, self-healing and acid-triggered release	[8,77,86,102]
Thiol–(meth)acrylate Michael addition and disulfide self-crosslinking	Physiological pH and temperature, no light or initiator; thiolation via carbodiimide chemistry with cystamine; disulfides form by spontaneous oxidation	Injectable, gelling in situ under physiological conditions; moderate to high strength, tunable with thiol content; moderate degradation, disulfides cleaved by intracellular glutathione	High: redox-responsive in the reducing tumor environment	Simple composition with no added crosslinker, but thiols oxidize during storage, so shelf-life, packaging, and oxygen exposure must be controlled	HA sub-network—alternative when redox-triggered release and nanoparticle dispersion are required	[18,54,98]
Photo-crosslinking of methacrylated polysaccharides	HAMA + CMCMA with LAP under 400 nm visible light, or HA-catechol + CMC-methacrylate with a photoinitiator under 365 nm UV	Applied as a liquid and solidified in the defect; high and tunable strength with the degree of methacrylation; slow to moderate degradation	Low, unless combined with a dynamic network	Photoinitiator residues and radicals require evaluation; visible light is preferable because melanin absorbs UV strongly in pigmented skin	Second choice for in situ application when the gel must be shaped in the surgical cavity; prefer visible light	[29,70,88,103]
Ionic and physical interactions	Simple mixing in aqueous medium; γ-irradiation (1 kGy/h, 20 h) used to obtain low-molecular-weight HA before blending	Mild and reversible gelation; low strength; fast degradation (hours to days) through ion exchange in exudate	Ion- and pH-responsive; Fe^3+^ can double as a Fenton catalyst for chemodynamic therapy	No chemical crosslinker to justify, but γ-irradiation degrades HA, which conflicts with radiation sterilization	Complementary rather than structural: use to load and stabilize nanoparticles and to preserve CD44 sites; can be weak if used alone for a post-surgical dressing	[13,85,101,104]

Conditions are those reported in the cited studies and are indicative; they have not been optimized for a CMC–HA–nanoparticle platform, which has not yet been built. The last column is a design recommendation of the present review. Abbreviations: CMCMA, methacrylated carboxymethylcellulose; LAP, lithium phenyl-2,4,6-trimethylbenzoylphosphinate.

**Table 4:** Comparison of nanoparticle classes reported for melanoma theranostics in the studies reviewed.

Nanoparticle Class (Examples in This Review)	Main Theranostic Function	Reported Performance	Imaging Capability	Biodegradation, Clearance and Main Toxicity Concern	Development Status	Ref.
Gold nanoparticles and nanorods	PTT through tunable NIR plasmon absorption; chemo-photothermal combination; biosensing	NIR-triggered release and chemo-photothermal killing in a cellulose-based hydrogel (colon cancer) and in an HA hydrogel targeting cancer stem cells; AuNP + NIR evaluated in melanoma	Photoacoustic and CT contrast; biosensing readout	Not biodegradable; clearance is size-dependent, with hepatic and splenic retention of larger particles; persistence of a non-degradable metal and dose-dependent accumulation	Most clinically advanced class of nanoparticle	[14,33,92,93,114,115]
Carbon dots	PTT with secondary ROS generation; intrinsic fluorescence	Photothermal conversion efficiency of 31.9% and tumor suppression in a B16F10 model when embedded in an HA hydrogel	Fluorescence imaging (visible to NIR)	Small dots are renally cleared, and the carbon core is metal-free; composition is heterogeneous between synthesis routes and long-term data are scarce	Preclinical	[24,77]
Iron oxide nanoparticles	Magnetic hyperthermia; Fenton-type chemodynamic therapy; drug carrier	Magnetothermal and chemodynamic therapy of cancer cells in CMC-based nanoarchitectures; peroxidase-like nanozyme activity	MRI (T2 contrast)	Biodegradable; iron enters physiological iron metabolism; iron overload and oxidative stress at high doses	Clinically established for imaging and hyperthermia	[42,61,116]
Cerium oxide (nanoceria)	Catalase-like nanozyme: sustained O_2_ generation and relief of hypoxia; ROS modulation via the Ce^3+^/Ce^4+^ couple	≈42% tumor-volume reduction and reduced HIF-1α when combined with Ce6 and CaO_2_ in a melanoma model	None reported	Not characterized; prolonged organ retention expected; redox activity is context-dependent (pro- or antioxidant), and clearance is unknown	Preclinical	[97]
Silver nanoparticles	Antibacterial action; antitumor activity through oxidative stress and apoptosis	Synergistic killing of A375 melanoma cells with doxorubicin and antibacterial activity in a CMC hydrogel, in which CMC acts as a reducing and capping agent	None reported	Dissolution to Ag^+^ with renal and hepatobiliary elimination; dose-dependent cytotoxicity of the released Ag^+^	Marketed in wound dressings	[25]
Calcium peroxide	In situ O_2_ and H_2_O_2_ generation for hypoxia relief and chemodynamic therapy	Improved antibacterial and phototherapeutic outcomes in HA-based systems for wounds and melanoma	None	Hydrolyses to Ca^2+^ and O_2_/H_2_O_2_; burst, uncontrolled hydrolysis with alkaline by-products that can destabilize the network	Preclinical	[5,97]
Manganese oxides and Mn^2+^	TME-responsive O_2_ generation and GSH depletion; Mn^2+^ as MRI agent and reversible crosslinker	MRI-guided chemo-photodynamic therapy as a coordination crosslinker; antibacterial and anticancer activity of Mn_3_O_4_ in a CMC hydrogel	MRI (T1 contrast)	Dissolution to Mn^2+^ with hepatobiliary elimination; hepatic and renal biochemical and histopathological alterations reported from 100 mg/kg	Preclinical	[78,96,100]
Cadmium-based quantum dots	Fluorescence imaging; stimulus-responsive carriers	Fluorescent CMC nanohydrogel for tumor theranostics, but in vivo tracking failed at the concentrations used	Fluorescence (visible to NIR)	Core degradation releases Cd^2+^, with size-dependent renal and splenic clearance; heavy-metal toxicity and regulatory phase-out in other application domains	Preclinical, with regulatory restrictions	[10,64,99,117]
Cadmium-free quantum dots (Ag-In-S, Ag_2_S)	NIR fluorescence bioimaging; nanocarrier platform	CMC-conjugated AIS/ZAIS quantum dots for bioimaging of cancer cells	Fluorescence in the NIR window	Not fully characterized, but no cadmium is released; the long-term fate of In and Ag is not established	Preclinical	[15,16,60,64]
Nanohydroxyapatite	Intracellular Ca^2+^ overload and ER stress; repolarization of tumor-associated macrophages	78.6% tumor inhibition in a B16F10 model when delivered from a thiolated HA hydrogel	None reported	Lysosomal dissolution into Ca^2+^ and phosphate; aggregation without surface modification and unknown durability of the immunomodulatory effect	Preclinical (the bulk ceramic is clinically established)	[98]

Values such as photothermal conversion efficiency are given only where the original study measured them, since they depend on particle size, shape, concentration and irradiation conditions and are not comparable across studies. Abbreviations: CT, computed tomography; ER, endoplasmic reticulum; HIF-1α, hypoxia-inducible factor 1α; MRI, magnetic resonance imaging; TME, tumor microenvironment.

## Data Availability

No new data were created or analyzed in this study. Data sharing is not applicable to this article, as this is a literature review with no primary/original datasets generated.
